# Optimizing anatomy dissection teams using the Yukari method: A peer compatibility‐based approach

**DOI:** 10.1002/ase.70124

**Published:** 2025-10-03

**Authors:** Tohru Murakami, Toru Araki, Yuki Tajika, Hitoshi Ueno, Sotaro Ichinose, Hirohide Iwasaki, Hiroshi Yorifuji

**Affiliations:** ^1^ Department of Anatomy Gunma University Graduate School of Medicine Maebashi Japan; ^2^ Faculty of Informatics Gunma University Maebashi Japan; ^3^ School of Radiological Technology Gunma Prefectural College of Health Sciences Maebashi Japan; ^4^ Department of Gross Anatomy Kyorin University Faculty of Medicine Mitaka Japan

**Keywords:** anatomy education, combinatorial optimization, dissection team assignment, learning outcome enhancement, local search algorithm, peer compatibility, team‐based learning, Yukari method

## Abstract

Human anatomy dissection serves as a cornerstone of medical education, fostering not only anatomical knowledge but also teamwork and professionalism. Given the considerable intellectual, physical, and emotional demands of dissection, effective team dynamics are essential for student success. To enhance learning experiences and academic outcomes, we developed the “Yukari method”—an automated system for optimizing anatomy dissection team assignments. This method uses a heuristic local search algorithm to maximize peer compatibility based on student peer preferences and motivation levels collected via a secure web survey. Compared to random and self‐selected teams, those assigned using the Yukari method showed approximately a 10% improvement in academic performance. Student satisfaction with Yukari‐assigned teams was significantly higher than with random assignment and comparable to self‐selection. This increased satisfaction, in turn, correlated with better academic outcomes. These findings suggest that the Yukari method is effective in medical education and potentially useful in other team‐based disciplines, such as engineering and social sciences.

## Graphical abstract

The Yukari method assigns dissection teams using peer compatibility and a local search algorithm. This approach improved student satisfaction and academic outcomes while reducing administrative burden.

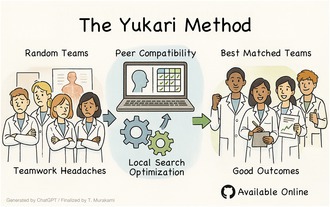

## INTRODUCTION

Human anatomy dissection has remained a core component of medical education in many countries, despite substantial curricular reforms over the past few decades.^1^, ^2^, ^3^, ^4^ Dissection offers students opportunities to develop professionalism, humanism, and teamwork as well as anatomical knowledge.^5^, ^6^, ^7^

Dissection has been conducted in small teams since the establishment of modern anatomy teaching in the 19th century,^8^, ^9^, ^10^, ^11^ as illustrated in the photo album *“Dissection: Photographs of a Rite of Passage in American Medicine 1880–1930”*.^12^, ^13^ This long‐standing practice predates the emergence of small‐team pedagogical methods, such as team‐based learning (TBL) and problem‐based learning (PBL), which have become widely adopted in medical education.^14^

Small‐team dissection is regarded as more beneficial than large‐group work for promoting hands‐on anatomical learning, peer teaching, and the development of social skills and humanistic values. Several recent studies have documented these advantages of small‐team dissection over large‐group settings.^15^, ^16^, ^17^

While teamwork and mutual support are key to success in dissection laboratories, students often experience high levels of stress due to academic demands, physical exhaustion, mental strain, and delicate social dynamics over the course of several months.^18^, ^19^, ^20^ These factors underscore the importance of effective team‐assignment practices and ongoing management of dissection teams.

### Team assignment methods in anatomy dissection

Team assignment methods commonly used in anatomy dissection laboratories include random or alphabetical assortment, student self‐selection, and teacher assignment based on student characteristics.^21^

Many institutions—particularly those employing TBL or PBL—adopt random or alphabetical grouping as the default team‐building method.^22^ For instance, Inuwa et al. applied TBL to their anatomy class by assigning small teams via random allocation.^23^, ^24^ Similarly, Burgess et al. and Huggett et al. ran TBL‐based dissection courses in which teams were formed alphabetically.^25^, ^26^

Another commonly used method is student self‐selection based on peer preferences. Johnson et al. investigated a multimodal, multidisciplinary anatomy course in which students were allowed to form their own teams.^27^

Teams can also be deliberately structured for diversity, balancing factors such as gender, personality traits, learning styles, academic ability, and prior experience. This approach aims to foster synergistic effects, or “chemistry,” within the team. For instance, Vasan et al. employed a modified TBL for medical gross anatomy and embryology, in which class coordinators assigned students to teams balanced by their backgrounds.^28^, ^29^

### Motivation and aims of the Yukari method

Our interest in developing an automated team assignment method for dissection laboratories was prompted by a significant challenge we encountered. From 1997 to 2013, students were allowed to form their own teams in the anatomy course. However, in 2014, a limited availability of human bodies delayed the finalization of the team size, necessitating random team allocation. This approach resulted in increased intra‐team conflicts, highlighting the need for a more efficient team assignment strategy to improve student satisfaction and academic performance.

In 2015, we developed a computer‐based system for optimizing team assignments, code‐named the “Yukari method.” The name was inspired by Yukari Nejima, a fictional character from the Japanese near‐future manga series “*Love and Lies*”.^30^ In the story, Yukari is a 15‐year‐old student living in a society where the government assigns marriage partners using compatibility scores based on genetic and social data. Additionally, “Yukari” in Japanese denotes a connection or relation, often implying a subtle or hidden bond.

The Yukari method was designed to match team assignments to peer‐to‐peer preferences and students' commitment to the course. Elements such as prior experience, academic ability, and learning style were deliberately excluded. The underlying assumption was that students themselves are best positioned to identify peers with whom they can learn most effectively. The method aimed to promote stronger peer relationships within teams and, consequently, enhance academic outcomes. Accordingly, we formulated the following hypothesis: that the Yukari method would improve both academic performance and student satisfaction compared with random assignment and self‐selection.

This report outlines the Yukari method and evaluates its impact on student performance and satisfaction in comparison with other approaches including random allocation and self‐selection. To our knowledge, this is the first report of its kind in the field of anatomy education.

## MATERIALS AND METHODS

### Participants and course setting

The gross anatomy course is taken in the second year (see below for the curricular background). Between 2012 and 2022, 119–139 students were enrolled annually (53%–71% male; Table 1). Most students entered directly after high school, and 15 transfer students joined each year. The gross anatomy course lasts 14 weeks and includes 41 h of lectures and 182 h of labs (2016 data). Students worked in small teams to dissect a donated human body using the Japanese version of “Grant's Dissector”.^31^, ^32^ Each team dissected the same body for the entire course.

**TABLE 1:** Participants and team assignment methods.

Year	2012	2013	2014	2015	2016	2017	2018	2019	2020	2021	2022
Number of students	126	131	129	139	135	126	134	125	126	119	126
*Student type (%)*											
Male	79 (62.7)	89 (67.9)	90 (69.8)	99 (71.2)	94 (69.6)	91 (72.2)	82 (61.2)	83 (66.4)	86 (68.3)	72 (60.7)	67 (53.2)
1st year repeater	1 (0.8)	3 (2.3)	2 (1.6)	4 (2.9)	4 (3.0)	3 (2.4)	5 (3.7)	2 (1.6)	1 (0.8)	11 (1.7)	7 (5.6)
Regular	104 (82.5)	106 (80.9)	102 (79.1)	106 (76.3)	108 (80.0)	104 (82.5)	107 (79.9)	105 (82.5)	110 (87.3)	106 (89.1)	109 (86.5)
Anatomy repeater	6 (4.8)	7 (5.3)	10 (7.8)	12 (8.6)	8 (5.9)	4 (3.2)	7 (5.2)	2 (1.6)	3 (2.4)	4 (3.4)	9 (7.1)
Transfer	15 (11.9)	15 (11.5)	14 (10.9)	15 (10.8)	15 (11.1)	15 (11.9)	15 (11.2)	15 (12.0)	16 (12.7)	15 (12.6)	15 (11.9)
Transfer repeater	0 (0.0)	0 (0.0)	1 (0.8)	2 (1.4)	0 (0.0)	0 (0.0)	0 (0.0)	0 (0.0)	1 (0.8)	0 (0.0)	0 (0.0)
*Team assignment*											
Team size	4	4	5	4	4	4	4	4	4	4	4
Number of teams	33	33	26	35	33	32	33	32	32	30	32
Team assignment method	Self	Self	Random	Yukari 1	Yukari 2	Yukari 2	Yukari 2	Yukari 2	Yukari 2	Yukari 2	Yukari 2
Yukari code version	–	–	–	1	1.1	2	2	2	2	2	2
Eligible for academic outcome analysis	Yes	Yes	Yes	Yes	Yes	Yes	Yes	No	No	No	No

*Note*: The number of participants and the methods used for team assignments from 2012 to 2022. It includes detailed information on student composition by type, the size and number of teams, the specific assignment methods employed each year, and eligibility for statistical outcome comparisons.

Team assignment methods varied by year (Table 1). In 2012 and 2013, students formed their own teams of four. In 2014, teams of five were assigned randomly. In 2015, the “Yukari method” was introduced (Yukari 1), and it was further refined in 2016 (Yukari 2; see following sections for details).

Donated human bodies used in the course were embalmed with a formalin‐based fixative containing 3.7% formaldehyde and a water‐soluble contrast agent. Further details of the fixation procedure are described in Murakami et al.^33^

### Assessment of peer compatibility in the Yukari method

Peer preference data were collected for Yukari 1 using a secure online survey (“Yukari Peer Preference Survey 1”) and administered before class. Each student rated all classmates on a five‐point Likert scale, with “5” indicating a highly desirable teammate and “1” an undesirable teammate. Blank responses were assigned a “3.” Prior to the survey, an information session instructed students to base their ratings on anticipated academic compatibility rather than personal preference. In 2016, two items—motivation toward the anatomy lab and willingness to stay late—were added to the survey for Yukari 2 (“Yukari Peer Preference Survey 2”; Appendix A1). Compatibilities between all possible student pairs were calculated from survey data using confidential matching matrices (Table 2). For 2015, peer preferences quantified with the “Peer Preference Matrix” determined peer compatibility (Yukari 1). For Class 2016 and later (Yukari 2), motivation and time match quantified with “Motivation–Time Matrix” were integrated as:

$$\begin{array}{c} \text{Peer compatibility}=\text{Peer preference}\\ +\text{Minimum of}\;\left(\text{Motivation match and Time match}\right) \end{array}$$

Calculations were performed in Apple Numbers (Apple Inc., Cupertino, CA), and results were exported as input files for “Yukari Code.”

**TABLE 2:** Compatibility matrices.

A: Peer preference matrix						B: Motivation–time matrix					
Response	1	2	3	4	5	Response	1	2	3	4	5
1	1	1	2	2	3	1	1	0	−1	−2	−3
2	1	2	2	3	4	2	0	0	−1	−2	−2
3	2	2	6	7	8	3	−1	−1	0	−1	0
4	2	3	7	9	10	4	−2	−2	−1	1	2
5	3	4	8	10	10	5	−3	−2	0	2	2

*Note*: These matrices were used to calculate peer compatibility scores: (A) Peer preference matrix based on students' survey responses; (B) Motivation–Time matrix incorporating students' reported motivation for dissection and willingness to stay late.

### Team‐assignment computer program “Yukari Code”

The team‐assignment program “Yukari Code” uses a heuristic local search algorithm in C++. The code was improved for speed and accuracy in later versions. The code, executables (for macOS and Windows), sample data, README files, and support tools for the local implementation of the Yukari method are available under the MIT license in the GitHub repository: https://github.com/tohru‐murakami/Yukari_Code_2.^34^ A summary of the algorithm steps and the source code is also given in Appendix A2.

### Examinations and assignments

Student performance was evaluated through a combination of written and practical examinations as well as team‐based assignments. Point allocations for each component were posted on the class blog (https://anatomy.med.gunma‐u.ac.jp/tag/scoring/; Table S1).

Students took three examinations, each consisting of a 1.5‐h written part and a 1.5‐h practical part. The written part included descriptive, fill‐in‐the‐blank, multiple‐choice, and short‐answer questions. The practical part comprised 33–50 stations featuring dissected human bodies, anatomical sections, skeletal specimens, and medical images. Each station contained two identification questions, and students rotated every 80 s.

Teams also completed mandatory projects in addition to the standard *Dissector* procedures, including anatomical sketches (e.g., brachial plexus, coronary arteries, and celiac artery), clinically oriented dissections (e.g., segmental bronchi and hepatic segments), and a report integrating postmortem radiology with dissection findings.^33^ Team‐based assignments were graded collectively, with all members receiving the same score.

### Surveys of student viewpoints regarding team assignments

Student viewpoints were assessed using the “Yukari Viewpoint Survey” (Appendix A3, Table S2). The survey included three items, each rated on a 1–5 scale: initial impression of the assigned team, final satisfaction with the team, and impression of the team assignment method. For the 2012–2014 cohorts, the survey was retrospective and voluntary, distributed in 2017. To encourage participation and minimize selection bias, small incentives were offered.

### Statistical analyses

For analysis, the 2012–2013 (self‐selected) and 2016–2018 (Yukari 2) cohorts were each combined. Classes from 2019 onward were excluded from statistical comparisons of academic outcomes due to personnel and curriculum changes.

Statistical methods were selected in accordance with best practice, taking data characteristics into account (Table S3; Petrie & Sabin).^35^, ^36^, ^37^, ^38^ Outliers were removed using Tukey's test for jitter plots.^39^ All analyses were performed with R (v 4.5.1; R Foundation, Vienna, Austria) and RStudio (v 2025.05.1+513; RStudio, PBC, Boston, MA).

### Educational background

As background to this study, the medical curriculum is outlined as follows. The medical program spans six years, with all medical science subjects required (Figure S1). Liberal arts and basic sciences are studied in years 1 and 2, while basic medical sciences are taught from years 1 to 3. Early clinical exposure in years 1 and 2 is provided through small‐group placements, with teams assigned either alphabetically or randomly. Anatomy is taught in the second semester of year 2. Figure S2 shows the timeline of the anatomy course with Yukari team assignment. From years 4 to 6, clinical medicine is taught through lectures, and clinical training is conducted in teams balanced according to academic performance.

### Security and ethical approval

Survey and team assignment data were managed confidentially by one faculty member and stored securely in encrypted form. Raw data were not shared. The use of the students' data in this research was approved by the Gunma University Ethical Review Board for Medical Research Involving Human Subjects (accession #2017‐106, effective through March 2023).

### Disclosure of AI assistance

All research, coding, and writing were done by the authors. English proofreading of the manuscript was assisted by ChatGPT (OpenAI, San Francisco, CA, USA). All content is solely the responsibility of the authors and was not generated by AI.

## RESULTS

### Impact of Yukari method on academic performance

**FIGURE 1:**
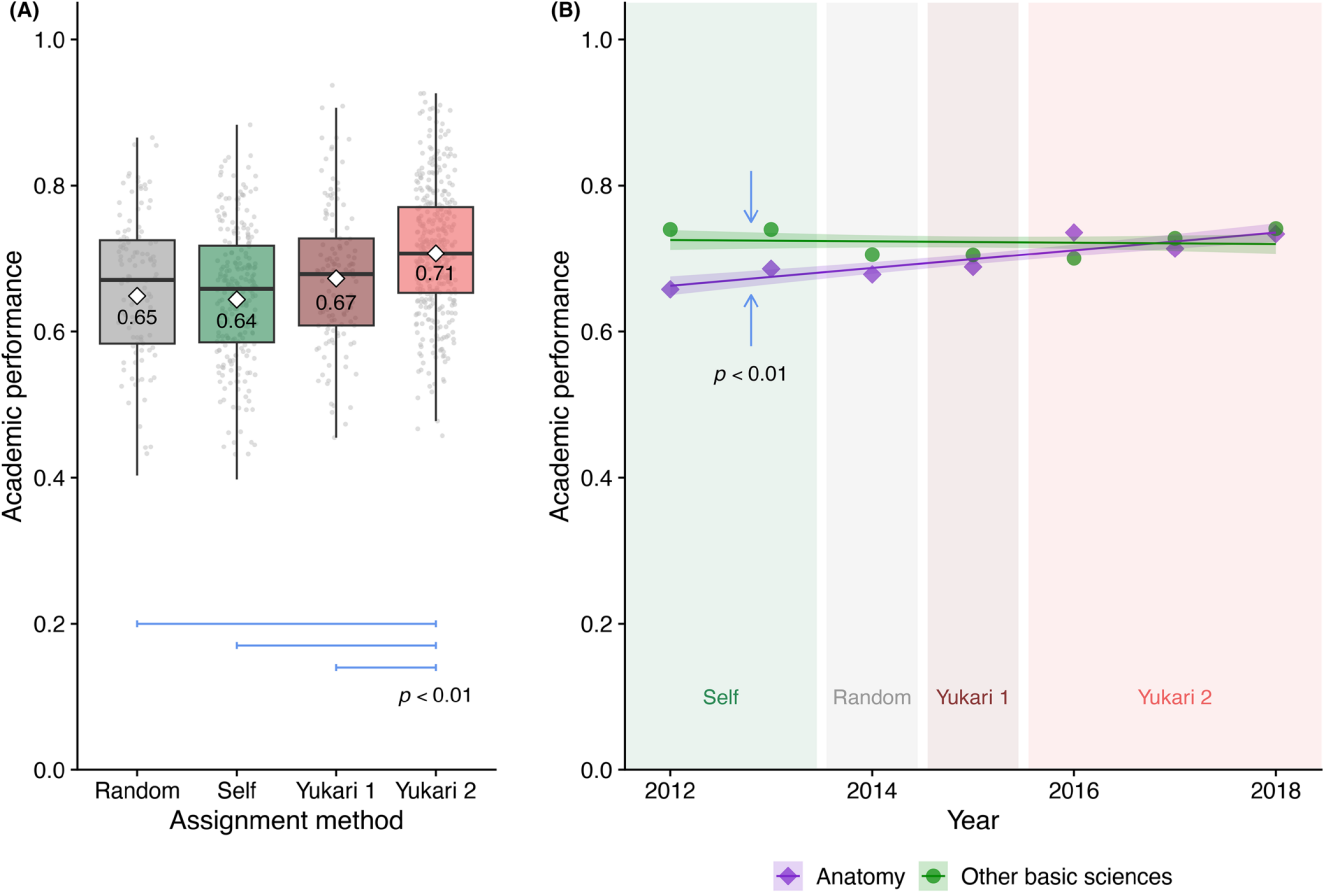
Impacts of team assignment methods on academic outcomes. (A) The academic performances of classes with different team assignment methods, including Random, Self, Yukari 1, and Yukari 2, are summarized in a box chart. The Tukey–Kramer multiple comparison test ^40^ indicated significant differences (*p* < 0.01) between Yukari 2 and the other methods (blue bars). The white diamonds and numbers denote means. (B) Comparison of yearly trends in academic performances between anatomy and other basic sciences combined (biochemistry, histology, neuroanatomy, systems physiology, and neurophysiology). ANCOVA showed a significant difference (blue arrows facing each other; *p* < 0.01) between the slopes of the regression lines with 95% confidence intervals.^41^

The Yukari method had a marked positive impact on academic outcomes. Students were categorized into four cohorts based on team assignment methods: random, self‐selected, Yukari 1, and Yukari 2. Among these, the Yukari 2 cohort achieved the highest performance, showing a 10% improvement compared to the other cohorts (Figure 1A). Since the method's introduction, anatomy scores have shown a clear upward trend. In contrast, performance in other basic sciences combined (biochemistry, histology, neuroanatomy, systemic physiology, neurophysiology) remained largely stable (Figure 1B), indicating minimal variation in overall academic strength across cohorts.

A multiple regression analysis of academic outcomes for Classes 2016–2018 (Yukari 2) showed significant positive associations with post‐course team satisfaction, available laboratory time, and either individual compatibility (average of each student's compatibility with other team members; not shown) or team compatibility (average of all compatibilities within the team) (Table 3). Motivation for dissection was not statistically significant, although it showed a positive trend. This may reflect the skewed distribution of responses, with only 10% of students scoring ≤3 and 90% scoring 4 or 5.

**TABLE 3:** Multiple linear regression analysis of academic outcomes.

Explanatory variables	Estimate	Std. error	*t* Value	*p*	2.50%	97.50%	Significance	VIF
(Intercept)	0.409	0.056	7.30	4.51E−12	2.99E−01	5.19E−01	***	
Satisfaction	0.016	0.005	2.90	4.12E−03	5.12E−03	2.66E−02	**	1.01
Motivation	0.018	0.009	1.93	5.49E−02	−2.86E−04	3.64E−02	.	1.36
Time	0.019	0.007	2.67	8.17E−03	5.06E−03	3.31E−02	**	1.36
Team compatibility	0.012	0.005	2.26	2.45E−02	1.60E−03	2.21E−02	*	1.01

*Note*: Significance codes: 0 ‘***’ 0.001 ‘**’ 0.01 ‘*’ 0.05 ‘.’ 0.1 ‘ ’ 1. Factors associated with academic performance in anatomy were analyzed using multiple linear regression analysis. The table presents the estimated coefficients (Estimate), standard errors (Std. Error), *t*‐values, *p*‐values, and 95% confidence intervals (2.50%, 97.50%) for each explanatory variable. Significance levels are indicated with asterisks, and the Variance Inflation Factor (VIF) is provided to assess multicollinearity. Values of VIF <10 suggest no serious multicollinearity.

**FIGURE 2:**
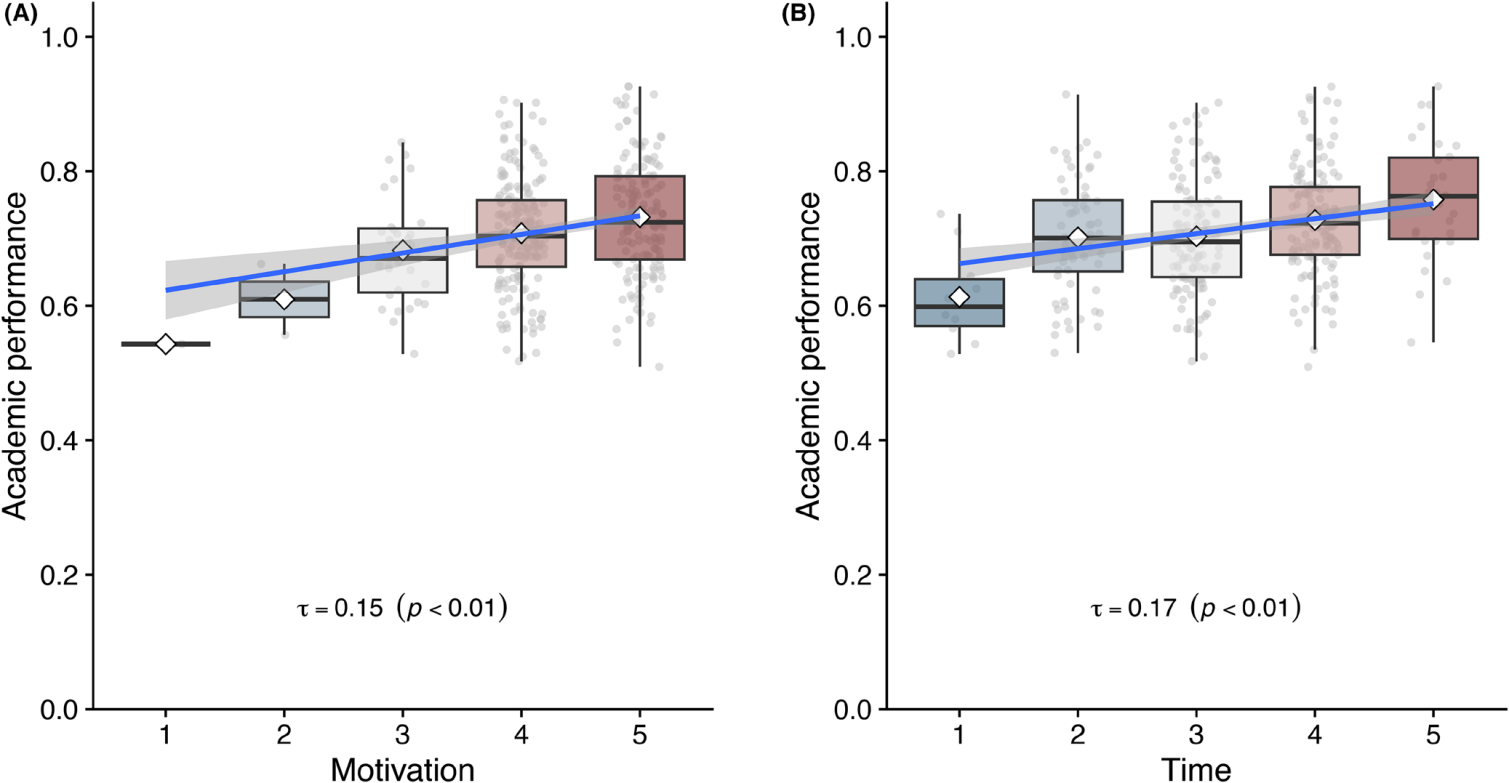
Correlations of motivation and time commitment with academic performance. Students of Yukai 2 were subdivided into five subgroups based on (A) motivation toward dissection and (B) willingness to stay late (time), each rated on a 1–5 scale (higher is better). Academic performance is summarized as box plots with jittered data points and superimposed regression curves (blue) with 95% confidence intervals. Kendall's *tau* indicated significant positive correlations in both cases (Motivation: *τ* = 0.15, Time: *τ* = 0.17; both *p* < 0.01).

**FIGURE 3:**
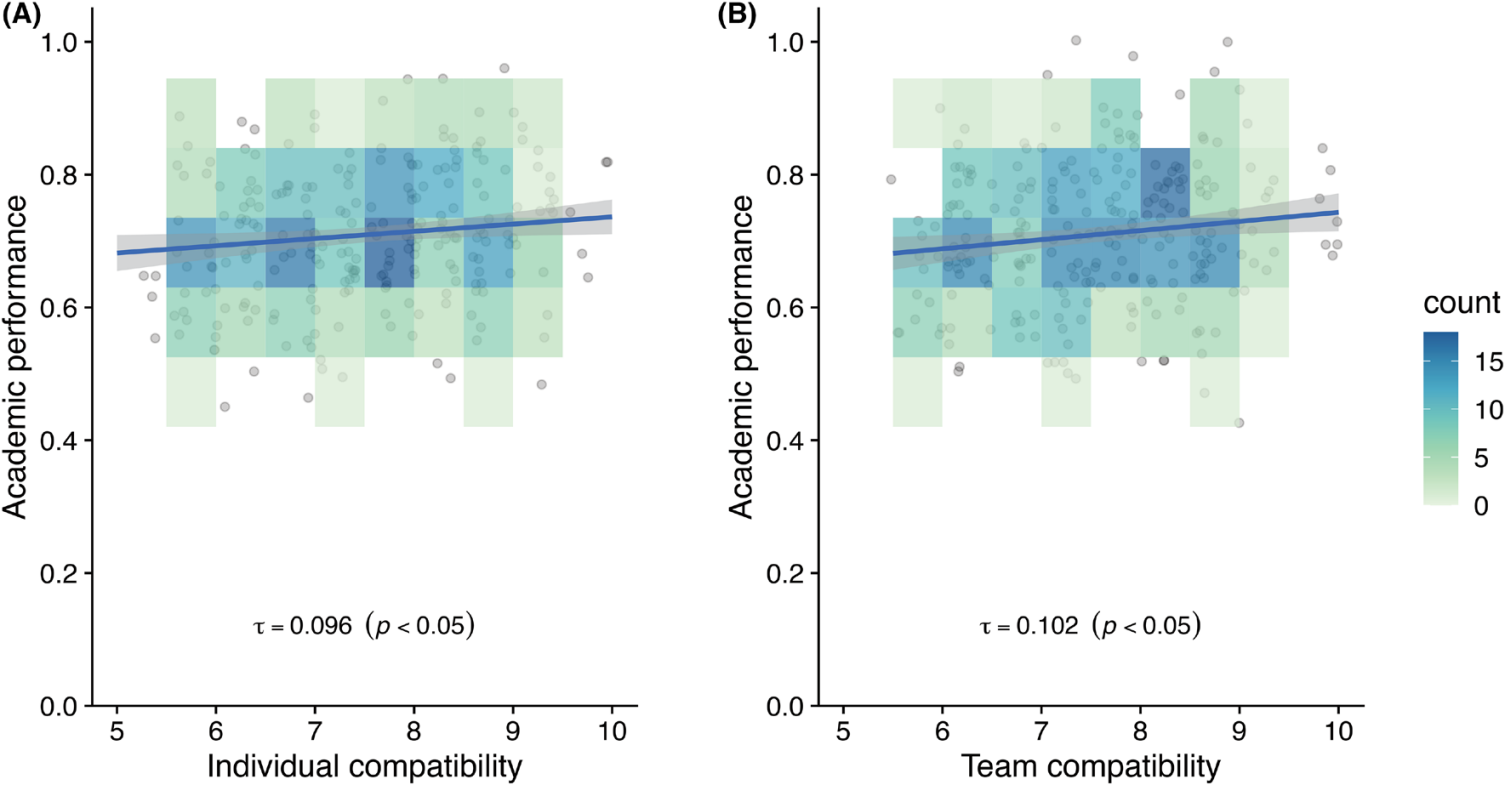
Impacts of compatibility on academic performance. Density plots showing the relationship between peer compatibility and individual academic performance in Classes 2017–2018, with compatibility scores ranging from 1 to 10. Compatibility is plotted as: A. Individual compatibility (average of a student's scores with teammates); B. Team compatibility (average of all pairwise scores within each team). Jittered data points are overlaid to illustrate data density. Regression curves with 95% confidence intervals are shown in blue. Kendall's *tau* ^42^ indicates a weak but statistically significant positive correlation in both panels (*p* < 0.05).

Additional analysis indicated that both motivation and time were individually correlated with academic performance (Figure 2). Simple linear regression also demonstrated significant positive relationships between academic performance and both individual and team compatibility (Figure 3).

**FIGURE 4:**
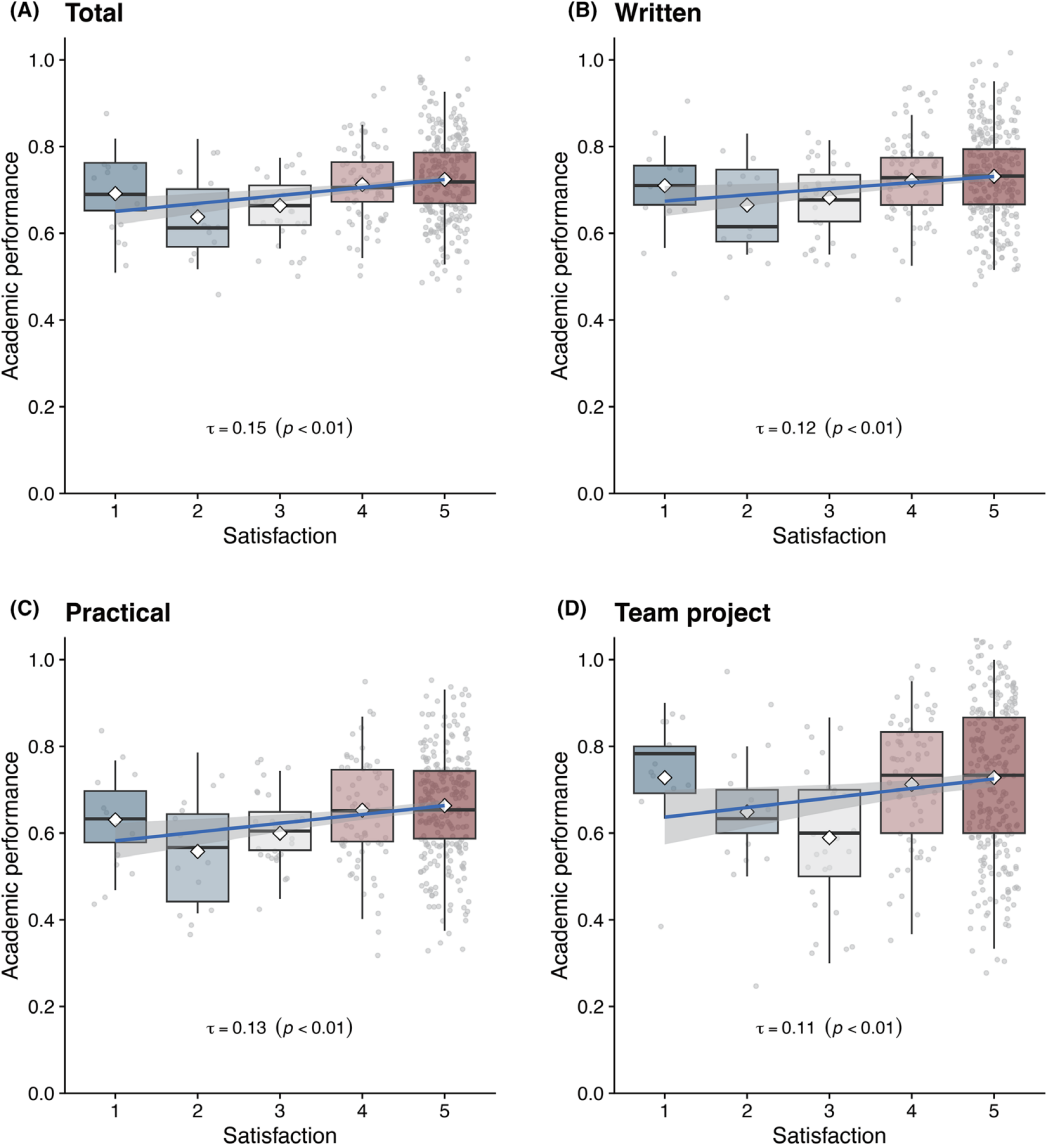
Impacts of satisfaction on academic performance across different modalities of performance evaluations. Students were subdivided into five subgroups based on their satisfaction with their own team (1–5; higher is better), and the academic performance ((A) Total, (B) Written examinations, (C) Practical examinations, (D) Team projects) of each subgroup is summarized as box plots with jitter plots superimposed. The blue lines represent regression curves with 95% confidence intervals. Kendall's *tau* indicated a significant positive correlation (*p* < 0.05) for each type of academic performance.

Students' satisfaction with their teams correlated positively with multiple outcome measures, encompassing both knowledge‐based self‐study and team‐based learning. To quantify this impact, Yukari 2 students were stratified into five subgroups according to satisfaction scores (1–5; details in the next section). Their academic outcomes (written tests, practical tests, team projects, and total scores) were plotted as box‐and‐whisker charts with superimposed regression lines and jittered points (Figure 4). The charts indicate that students' satisfaction was positively associated with both team‐based learning (practical examinations and team projects) and individual study (written examinations).

### Students' viewpoints regarding team assignments

To assess students' viewpoints regarding their team assignments, a separate survey was administered. Students were asked to rate the following aspects on a 1–5 Likert scale (where higher scores indicate more favorable responses)

1. Initial impression of their own team when the assignment was announced (Initial impression).
2. Viewpoint regarding their own team after the laboratory was completed (Satisfaction).
3. Viewpoint regarding the team assignment method of their class (Viewpoint to method).

It should be noted that the survey was administered retrospectively (1–4 years after the anatomy class) for Classes 2012–2014 and had lower response rates compared to Classes 2015–2022, in which the survey was administered immediately after completion of the anatomy course (Table S2).

**FIGURE 5:**
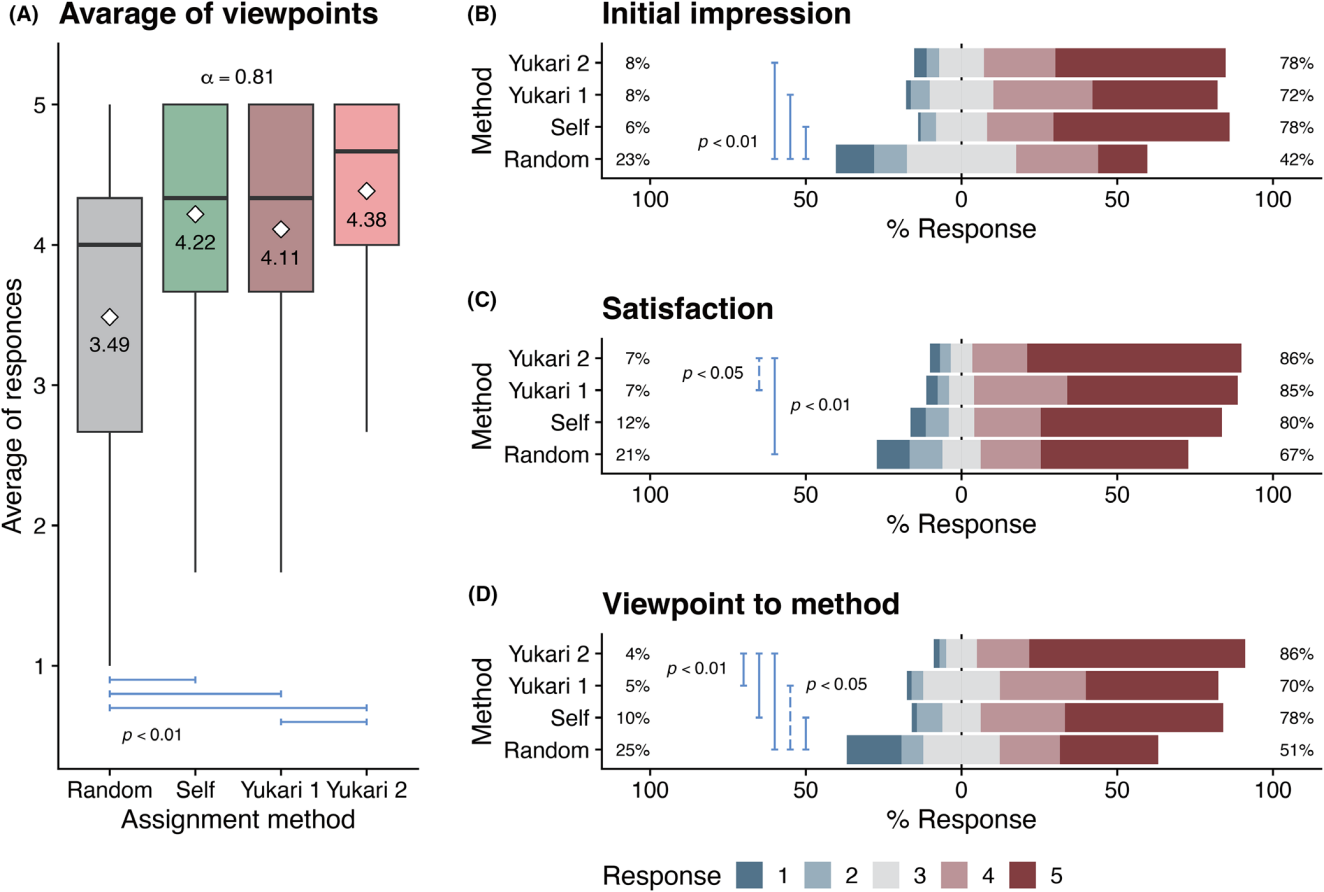
Students' viewpoints regarding team assignment. (A) The students' viewpoints (1–5 Likert scale; higher is better) regarding their own team before the laboratory started (Initial impression) and after the laboratory had concluded (Satisfaction), along with their team assignment method (Method), were combined as single values (Averaged responses) and summarized in a box chart. The blue bars show significant differences as indicated by the Tukey–Kramer test. Cronbach's *alpha* coefficient indicated internal consistency. The white diamonds and numbers denote means. (B–D) Individual responses for initial impression (B), satisfaction (C), and viewpoint on the method (D) were summarized as Likert‐type bar charts. The bars indicate significant differences as shown by the Steel–Dwass test.^43^, ^44^

**FIGURE 6:**
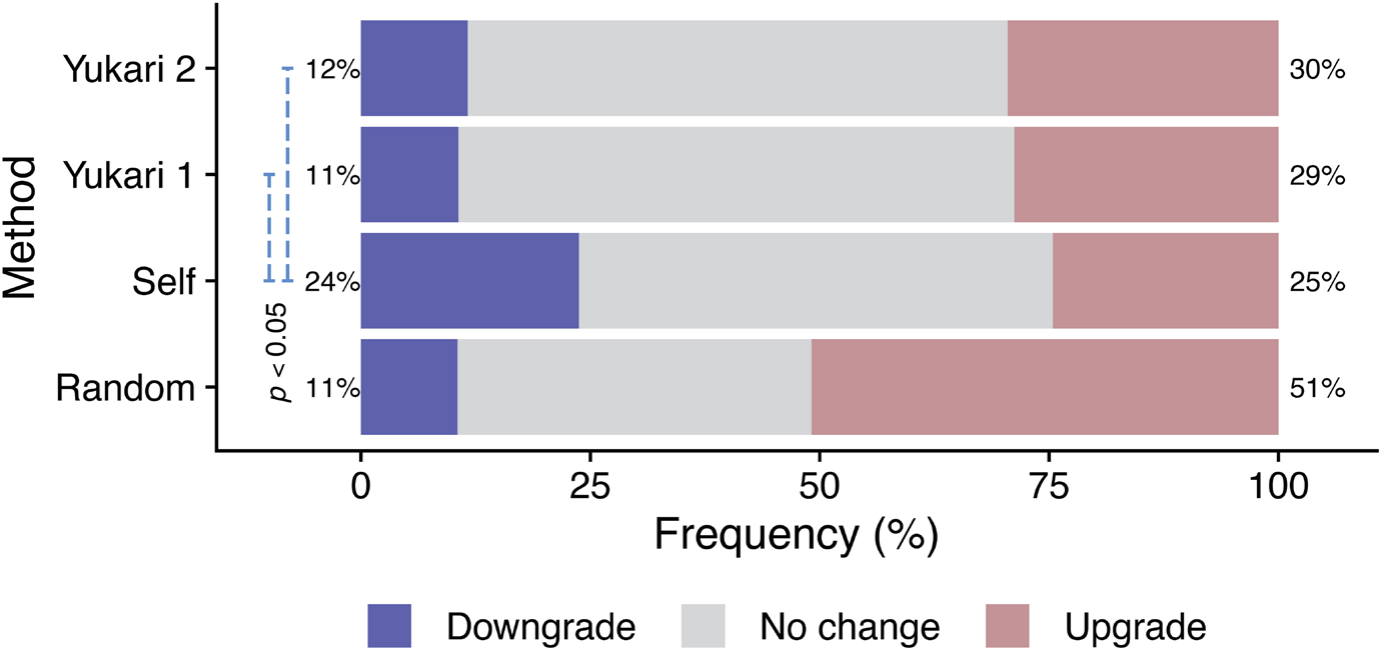
Changes in viewpoint regarding their own team after the laboratory was completed. The distributions of changes in students' viewpoints regarding their own team before and after the completion of the laboratory were compared between team assignment methods. The bars show significant differences (*p* < 0.05) as indicated by the Chi‐squared test, with *p*‐values adjusted using the Benjamini‐Hochberg method. The numbers represent *p*‐values. Blue = downgrade; Gray = no change; Brick red = upgrade. Note that the downgrade rate of self‐assigned teams was more than double that of the others, and the upgrade rate of random assortment was double that of the others.

As expected, the initial impression of their own team was markedly higher in self‐assignment and the Yukari method than in random assortment (Figure 5). These ratings increased significantly after the laboratory for random assignment, Yukari 1, and Yukari 2. Interestingly, for self‐assignment, students' ratings decreased after the laboratory. To clarify this point, we compared the distributions of before–after changes in viewpoints (Figure 6). In the self‐assignment cohort, roughly one‐quarter of students significantly downgraded their rating of their own team—twice the proportion seen with the other methods. The ratings regarding the team assignment method were markedly higher in self‐assignment, Yukari 1, and Yukari 2 than in random assortment.

**FIGURE 7:**
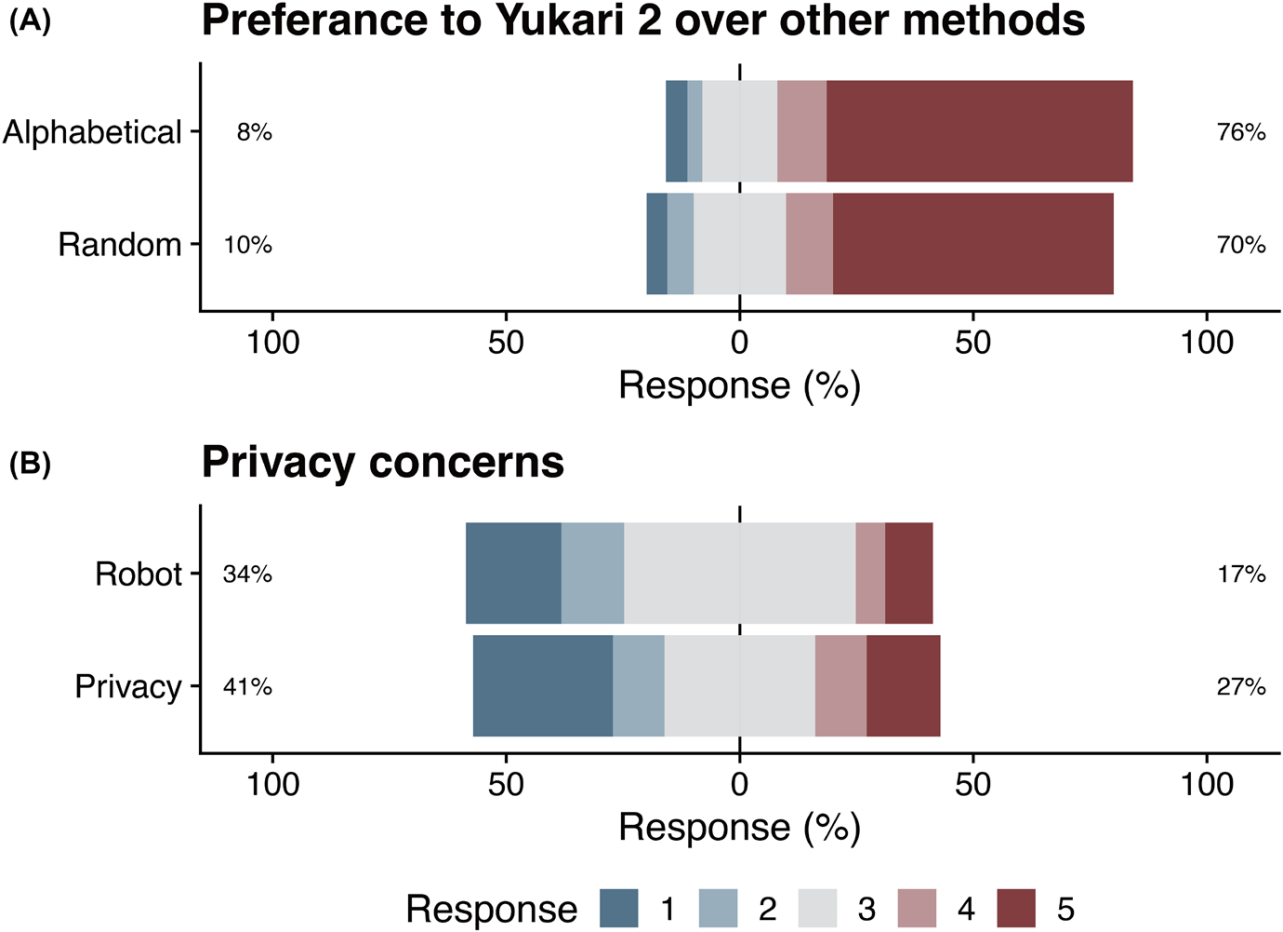
Students' preferences for Yukari and privacy concerns. (A) The students' preferences for Yukari 2 over random assortment and ID sorting on a Likert scale (1–5; higher means preferring Yukari better) were summarized as box plots. (B) The students' concerns about the privacy issues of Yukari 2 (1–5; higher means feeling safer) and their preference for a fully robotic system (1–5; higher scores indicate a stronger preference for the robot system) were summarized as Likert plots.

Students preferred the Yukari method to either alphabetical or random assortment, although some expressed privacy concerns about its implementation (Figure 7). To probe these issues further, four additional items were included in the Class of 2018 survey—two comparing assignment methods and two addressing privacy. The students had previously experienced alphabetical and random team assignments in other courses:

1. “Yukari is better than alphabetical assignment”
2. “Yukari is better than random assortment”
3. “A complete robot‐based anonymized team assignment system without human intervention would be favorable”
4. “Concerns about privacy issues with the current Yukari method”

More than 70% of students favored Yukari over alphabetical or random assignment methods. However, privacy concerns were noted—around 20% supported a fully automated robot‐based system, and nearly 30% expressed reservations about the current Yukari method.

## DISCUSSION

This study described the Yukari method, a computerized approach for optimizing dissection team assignments based on peer compatibility. The findings support our initial hypothesis that, compared with random assignment and self‐selection, the Yukari method enhances student outcomes. To our knowledge, this is the first report on automated optimization of team assignments in anatomy education that concurrently evaluates student viewpoints and academic outcomes.

Relative to these conventional methods, the Yukari method yielded positive results in efficiency, equal opportunity, and student self‐determination, as well as in student viewpoints and academic performance (Table 4). Specifically, the first version based solely on peer preference (Yukari 1) increased student satisfaction, whereas the refined version incorporating both peer preference and commitment factors (Yukari 2) improved not only student satisfaction but also academic performance, thereby outperforming Yukari 1.

**TABLE 4:** Comparison of team assignment methods in this study and previous works.

Methods	Random or alphabetical	Student selected	Balanced	Yukari
Respect for students' self determination	Low	High	Low	High
Students' equal opportunity	High	Fair	Low	High
Students' psychological comfort during team selection	High	Low	High	High
Efficiency of assignments	High	Low	High	High
Computational load	Low	Low	High	High
Students' initial impression	Low	High	Fair	High
Students' satisfaction	Fair	High	Fair	High
Students' lack of regret	High	Low	High	High
Academic outcome	(Baseline)	Insignificant—High	Insignificant—High	High

*Note*: Compares different team assignment methods used in our study and previous works, evaluating various factors such as respect for students' self‐determination, equal opportunity, psychological comfort during team selection, efficiency of assignments, computational load, students' initial impression, satisfaction, lack of regret, and academic outcomes.

The Yukari method ensured equal opportunities in team assignments while maintaining students' high motivation for the class. Motivation is a central driving force for learning, and such motivation is known to be influenced by students' self‐determination.^45^ A favorable team atmosphere in self‐managed teams has been shown to have significant impacts on academic outcomes.^46^ Such a favorable atmosphere in the classes using the Yukari method might explain its impact not only on team‐based learning (practical examinations and team projects) but also on individual studies (knowledge‐based written examinations).

The Yukari method could have reduced the effects of social skills on team assignments. A successful self‐selection for an individual student may depend on their social skills. Indeed, one quarter of the students in classes with self‐selection downgraded their viewpoints on their own team after the completion of the lab, which was twice the rate for those classes using the Yukari 1 or Yukari 2. Additionally, the proportion of open‐ended responses expressing psychological stress related to team assignment was 2–5 times higher in the self‐selection group compared to the other methods (detailed data are not disclosed due to privacy considerations).

The evaluation of peer compatibility can be modified to include other parameters. The rationale for employing peer preference as the core parameter was to improve the students' learning experiences, and hopefully also outcomes, under the conditions of high physical and mental stress in the dissection laboratories. Opportunities for real‐world team management (as in random assortment) or social chemistry (balanced assignment) were left to other team learning activities in our curriculum. Even so, the Yukari method could integrate features from other team assignment approaches by adjusting the peer compatibility matrices. For instance, teams could be balanced by academic ability, pairing a stronger student with a weaker one to increase their peer compatibility. This adaptability would make the Yukari method applicable to other subjects or institutions that employ small‐team learning, accommodating varying requirements and criteria for team assignments.

### In‐team diversity and “gunners”

In‐team diversity had a marked effect on academic performance. Across all assignment methods, two constraints were generally applied and coded in the “Yukari Code”: (1) no more than one transfer student per team, and (2) either two or no female students per team, although exceptions occasionally occurred.

**FIGURE 8:**
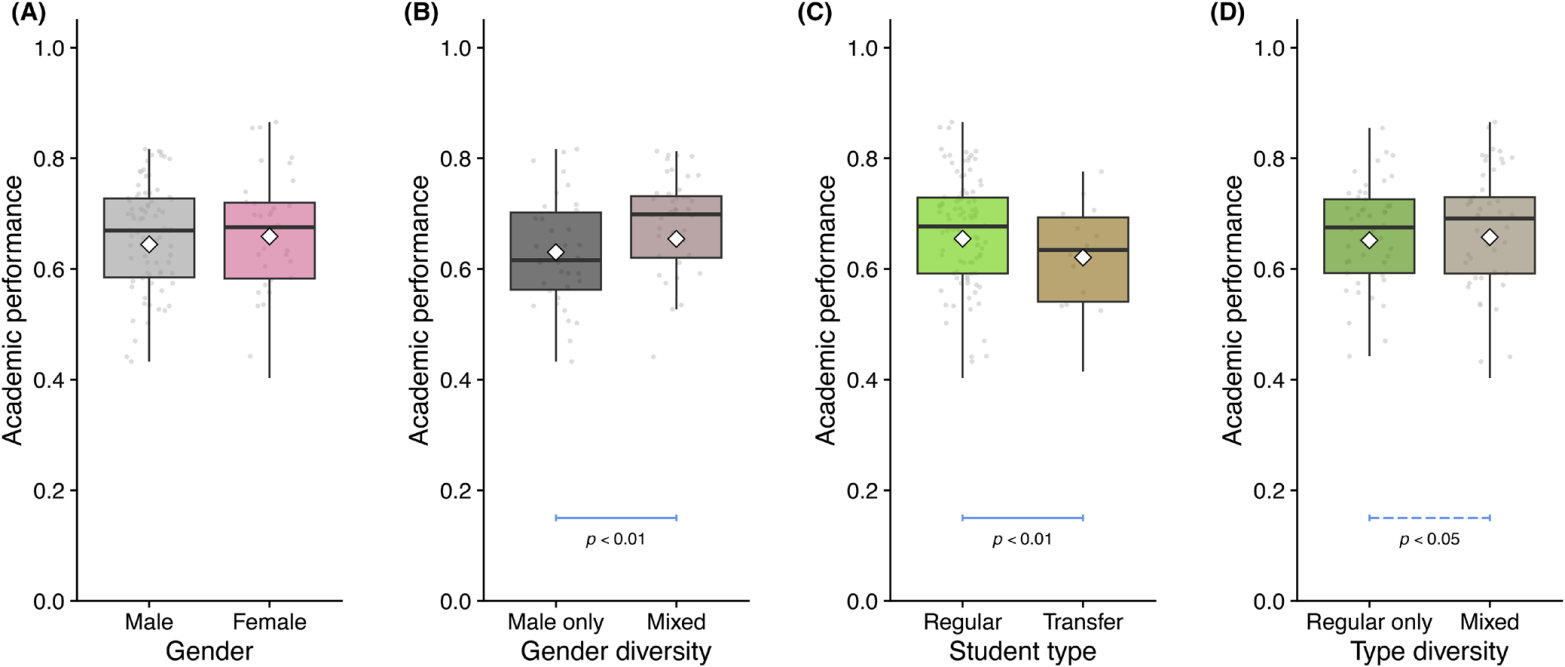
Impacts of gender balance and previous experiences on academic performance in class 2015 (random assortment). Students were subdivided by their gender (A), gender diversity in teams (B), student type (previous experience) (C), and diversity of previous experiences in teams (D), and the academic performance was summarized as box plots. Note that the balanced teams performed better. Bars show significant differences as indicated by the Tukey–Kramer test.

In Class 2015 (random assortment), teams with mixed characteristics (e.g., mixed gender, or a mix of regular and transfer composition) consistently outperformed more homogeneous teams (Figure 8). Similar patterns were observed in classes using other assignment methods, although these observations may be subject to bias due to interventions in the team assignment process (data not shown). Nevertheless, these findings support the importance of incorporating diversity considerations into the Yukari method.

Interestingly, the correlation between team satisfaction and academic outcomes (Figure 4) showed that several students with low satisfaction nevertheless achieved high academic scores. Although further investigation is warranted, this may suggest the presence of “gunner” students—individuals with strong academic performance but limited social compatibility.^47^

### Comparative evidence on team assignment strategies

This section reviews prior studies on how various assignment methods influence academic performance, student satisfaction, and team dynamics, thereby contextualizing the outcomes observed with the Yukari method.

Each assignment method carries distinct advantages and disadvantages, and a single best practice has yet to be established.^48^ Several studies have reported that student self‐assignment exerts a more beneficial impact on outcomes than random assortment.^49^, ^50^ Mushtaq et al., for example, conducted a quantitative study in a business‐management course that allowed students to select their own teams. Their findings revealed positive correlations between students' perceptions of various team characteristics and both their academic performance and group outcomes.^50^ Conversely, Oakley et al. found that stronger students clustered together, leaving weaker peers isolated—a configuration that proved counterproductive.^51^

Muller reported that teams balanced for academic strength enjoyed a modest student‐perceived advantage over randomly formed groups.^52^ Likewise, Hassaskhah and Mozaffari compared student‐selected groups with groups balanced by learning styles. They found that the balanced groups noticeably outperformed the student‐selected groups in terms of academic outcomes.^53^ Zheng et al. and Lambić et al. showed that balanced groups led to marked improvements in academic outcomes compared to random and self‐selected groups.^54^, ^55^ Adding support to these studies, Zhan et al. conducted an investigation of the effects of gender balance on students' team performance in computer‐supported collaborative learning. Their findings demonstrated that teams composed of two males and two females (2M2F) or four females (4F) outperformed other configurations.^56^ More recently, Bergtold et al. found that balanced teams based on academic strength outperformed others on early assignments, while self‐selected teams performed better later in the course, likely due to greater group cohesion.^57^

By contrast, several studies have documented mixed results for balanced assignments. For example, Huxham and Land detected no outcome differences between randomly formed groups and those balanced by learning style.^58^ Dillon and Cheney compared instructor‐assigned balanced teams with student‐selected teams and observed more intra‐team conflict in the former.^59^ Hilton and Philips compared three methods of team assignment—student selection, random assortment, and balanced assignment—in a financial accounting course. They found better experiences with student‐selected teams, although no differences were found in terms of team outcomes.^60^ Likewise, Pociask et al. and Farland et al. also reported no significant differences between these team assignment methods in terms of academic outcomes.^61^, ^62^

Collectively, the evidence suggests that no single method suits every student: Smith and Spindle found high‐ability students performed better in homogeneous teams, whereas low‐ability peers benefited from heterogeneity.^63^ This principle informed the Yukari method philosophy—“each student knows best with whom they learn best.”

### Computational challenges in team assignment

The task of assigning students to teams for team‐based learning becomes increasingly computationally intensive as the class size grows. Attempting to optimize team combinations can render the task practically infeasible. For example, consider partitioning 120 students into 30 teams of 4 members for optimization (Figure S3).

The naïve algorithm enumerates and evaluates all possible combinations by brute force. The number of possible combinations can be calculated as follows:

1204·1164·1124⋯4430!=9.89456⋯×10124

Even a TOP500 supercomputer (top500.org; June 2025) such as El Capitan at the Lawrence Livermore National Laboratory in California, USA, with a peak speed of 1.74 *EFlop/s* (1.74 × 10^18^ floating‐point operations per second) would require:

$$1.8\times 10^{99}\;\text{years}$$

to exhaustively search this space. This far exceeds the age of the universe (1.38 × 10^10^ years).

Local search, as implemented in the Yukari Code, is a heuristic approach that avoids exhaustive enumeration. Instead, it explores the solution space iteratively, with the goal of identifying local improvements that gradually enhance the overall solution. Its total complexity can be expressed in Big *O* notation as follows:

$$O\left(T\times n^{2}\right)$$

where *n* denotes the number of students and *T* the number of local‐search iterations (typically 10,000). For *n* = 120 and *T* = 10,000, the complexity is approximately:

$$O\left(10,000\times 120^{2}\right)=O\left(1.44\times 10^{8}\right)$$

This is vastly more efficient than brute‐force search and completes within seconds to minutes on a consumer‐grade computer (*TFlop/s* scale).

Automated or computer‐supported team assignment optimization has been reported in various fields and educational platforms. However, to our knowledge, there have been no reports on the development and application of automated team assignment systems for medical education.

Cavanaugh et al. developed a web‐based team assignment optimization system called “Team‐Maker” for their software engineering course. This system helped instructors conduct surveys to determine student characteristics and optimize team assignments using a heuristic algorithm based on teacher‐defined criteria. The entire process, from creating a survey to team assignment, could be completed in 1 h.^64^ Wang et al. developed a computer‐aided team assignment system called “DIANA” for their computer science class. This system was designed to create teams that exhibit internal diversity and external equality with other groups in terms of a maximum of seven student characteristics. DIANA resulted in greater student satisfaction and better team outcomes than random groups.^65^

In addition, a considerable number of team assignment systems have been developed featuring different algorithms. However, their effects on learning outcomes are often not thoroughly examined. For example, Hwang et al. and Moreno et al. developed an assistant system for organizing learning teams using a genetic algorithm.^66^, ^67^ Hübscher also developed a team matching system based on multiple criteria with a heuristic search algorithm.^48^ Zheng et al. and Sánchez et al. employed a genetic algorithm to assign teams balanced in terms of skills and peer preferences, showing improved academic outcomes in their computer programming classes.^55^, ^68^

### Robust optimization of dissection teams by the Yukari method

**FIGURE 9:**
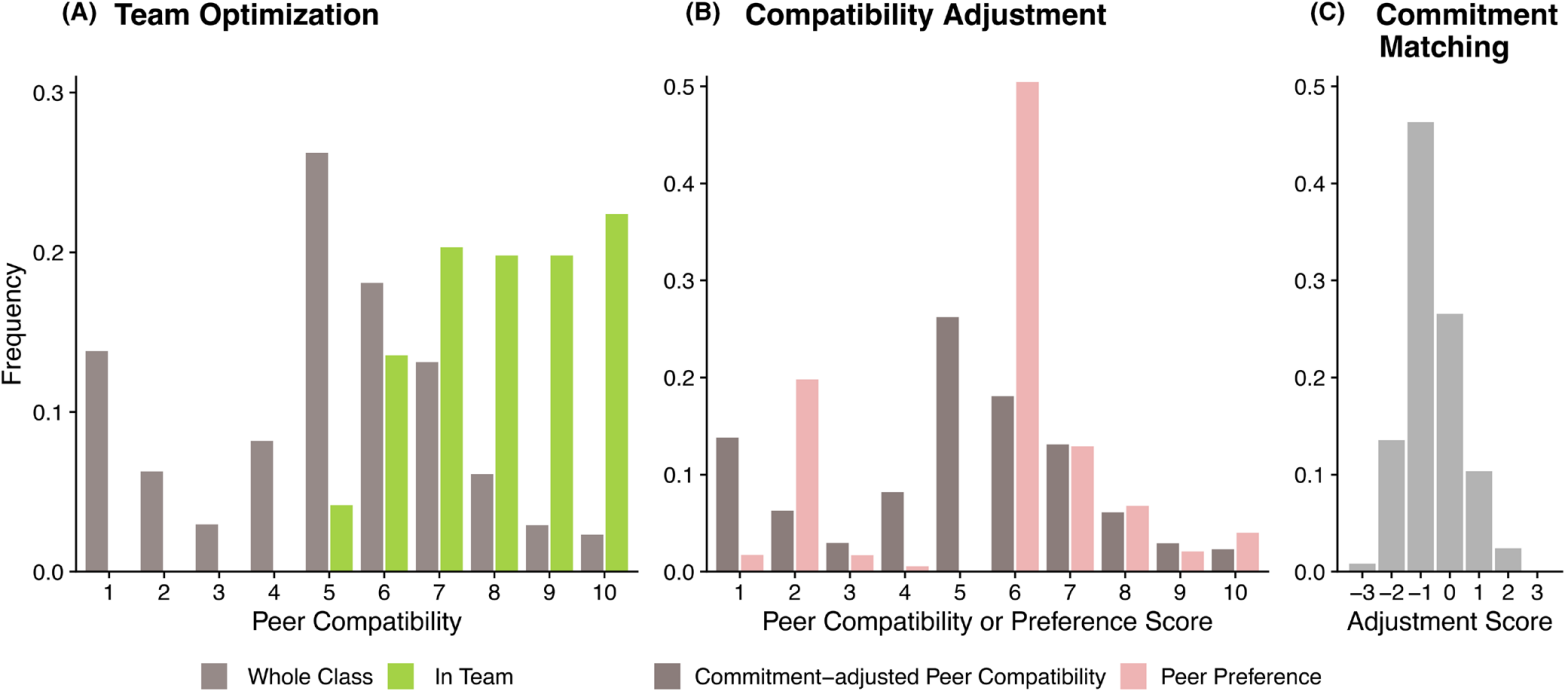
Distributions of peer‐to‐peer compatibility in the whole class and within the teams of class 2018 (Yukari 2). (A) Histogram of peer‐to‐peer compatibility scores in the whole class (8128 pairs, gray) and within the optimized teams (192 pairs, green). Note the successful elimination of incompatibilities (≤4) and the increase in favorable pairs (≥7). (B) Adjustment of peer‐preference scores (pink) by commitment matching to define peer compatibility (gray). (C) Distribution of commitment matching scores.

While the primary outcomes of this study concerned improvements in student performance and satisfaction, the Yukari method itself also demonstrated robustness in practice. Its core technology—the Yukari Code—employs a heuristic local search algorithm capable of processing large classes (>120 students) and generating optimized assignments within minutes on a standard iMac. The Yukari Code increased the number of favorable pairs within teams by effectively resolving peer incompatibilities (Figure 9A). The entire process—including data cleaning and compatibility calculations—could be completed by a single faculty member within one to two business days.

### Benefits of incorporating commitment factors into peer compatibility in Yukari 2

The positive correlations of commitment factors with academic outcomes may help explain why Yukari 2, which combined peer preference with commitment matching, outperformed Yukari 1. This improvement likely reflects, at least in part, greater accuracy in assessing peer compatibility.

As shown in Figure 9B, the distribution of peer‐preference scores displayed a sharp peak at the value of 6, reflecting reciprocal ratings of 3 from both students. Many of these ratings of 3, however, represented “undetermined” choices (e.g., “I do not know this person”) rather than genuine intermediate preferences indicating neutrality. Such undetermined responses may have introduced noise into the team assignment process. Incorporating commitment factors (Figure 9C) provided an independent dimension that helped compensate for these “unknown” pairs, thereby enhancing the robustness of Yukari 2, particularly under the constraints imposed by COVID‐19 restrictions, as detailed in the following section.

In addition, incorporating two distinct dimensions—peer preference and commitment matching—expanded the informational basis for evaluating peer compatibility. This increased the effective resolution of the optimization process and likely enhanced the accuracy of team assignments.

### Impacts of the COVID‐19 pandemic on students' viewpoints

The COVID‐19 pandemic led to major changes in anatomy education worldwide^69^, ^70^ and limited opportunities for in‐person peer interaction. This created a unique context to test the Yukari method, which relies heavily on students' familiarity with their peers. In Japan, the pandemic necessitated a shift to online lectures in 2020 and 2021; however, in‐person dissection remained possible during the fall–winter anatomy course, as the regional epidemic had largely subsided. Students in the Class of 2020 had prior in‐person interactions during their first academic year, whereas those in the Classes of 2021 and 2022 had fewer such opportunities. Despite these restrictions, survey data from 2020 to 2022 indicated that students reported high satisfaction with their teams (Figure S4).

### Limitation of the study

This study emerged from the need to address unforeseen challenges, requiring us to implement the most rational solutions under the circumstances. As a result, the methodological precision characteristic of a pre‐planned, statistically rigorous experiment was inevitably compromised. Restarting the study as a new trial was neither ethically nor technically feasible, as prospective students would likely gain prior knowledge of the Yukari method's advantages through hearsay from their seniors. Despite these limitations, we believe the findings provide valuable insights into the broader domain of anatomical and medical education. We sincerely hope that this study will serve as a catalyst for more sophisticated and methodologically rigorous investigations by educators and researchers at other institutions.

Team assignment based on peer compatibility, as in our Yukari method, is highly dependent on the students' appreciation of their classmates' characteristics. As our students learn anatomy during the second semester of the second year, they have one and a half years of previous opportunities to communicate with their peers. They also have two short courses of interprofessional team training before anatomy, where they receive training in teams at hospitals and geriatric health facilities. When students lack prior knowledge, a series of short‐term activities in temporary teams,^71^ or a random trial period^72^ can provide valuable insights into relationships with classmates, which would be useful for forming effective teams.

### Future directions and implications

A double‐blind randomized controlled trial within a single class could provide stronger evidence for the causal effects of the Yukari method. Preliminary power analysis showed that with 120 students and a standard deviation of 10% (from recent cohorts), a two‐sample *t*‐test could detect a 5‐percentage‐point difference in means with 80% power. Although this design is only marginally feasible statistically, it remains attainable. A practical challenge would be maintaining blinding, as students' awareness of their own peer relationships makes it difficult to fully mask group assignments.

While the current Yukari method requires manual handling of peer preference data, automation could further improve privacy protection. We propose a fully automated, web‐based system that securely encrypts data and automates surveys, data management, and team formation without human involvement. This system could be virtualized and made widely accessible.

Future research could enhance team optimization based on peer compatibility by incorporating additional parameters such as academic strengths, learning styles, and team roles. Leveraging the extensive dataset already collected from anatomy team formation, AI models could be developed to further refine peer compatibility evaluations within the optimization process. Additionally, investigating the long‐term effects on professional development and teamwork skills remains an important area for further study.

The Yukari method has not yet been tested outside anatomy education. However, its design makes it readily adaptable to various disciplines, such as clinical medicine, engineering, and the social sciences, that use collaborative learning. Applying the Yukari method in other educational settings could enable further evaluation and provide new insights into its utility and effectiveness.

### Significance of the study

This study advances anatomy education by presenting a novel and efficient method for team assignment. Our positive outcomes highlight the Yukari method's adaptability to other educational contexts, thereby enhancing learning experiences and academic performance.

## CONCLUSIONS

This study aimed to optimize team assignments in anatomy dissection using the Yukari method, a computerized approach based on peer compatibility. The method achieved the study's objectives by improving both academic outcomes and student satisfaction, while also minimizing administrative burden. These findings suggest that the Yukari method provides an effective framework for collaborative team formation, with potential applications in various areas of medical education and in other disciplines such as engineering and the social sciences.

## AUTHOR CONTRIBUTIONS

**Tohru Murakami:** Conceptualization; data curation; formal analysis; investigation; methodology; project administration; resources; software; writing – original draft; visualization; validation; writing – review and editing. **Toru Araki:** Writing – original draft; methodology; writing – review and editing; software; validation. **Yuki Tajika:** Conceptualization; data curation; writing – review and editing; resources. **Hitoshi Ueno:** Data curation; conceptualization; writing – review and editing; resources. **Sotaro Ichinose:** Data curation; writing – review and editing; resources. **Hirohide Iwasaki:** Data curation; supervision; project administration; writing – review and editing; validation; funding acquisition; resources. **Hiroshi Yorifuji:** Conceptualization; data curation; supervision; project administration; writing – review and editing; validation; funding acquisition; resources.

## ETHICS STATEMENT

The use of the students' data in this research was approved by the Gunma University Ethical Review Board for Medical Research Involving Human Subjects (accession #2017‐106, effective through March 2023).

## Supporting information


**Appendix A1.** Yukari peer preference survey 2. This appendix provides the full text of the peer preference survey (version 2), which was administered to all anatomy students via a secure Google Form. It was designed to collect ratings of students’ commitment to the dissection lab and their anticipated peer preferences for use in team optimization.

**Appendix A2.** The Yukari Code 2.0 and related files. This appendix provides the source code, executables, sample data, and supporting files of the Yukari Code 2.0 program, which implements the heuristic local search algorithm for team optimization. These files are included as Supplementary Materials and are available for replication or local implementation of the Yukari method.

**Appendix A3.** Yukari viewpoint survey. This appendix provides the full text of the Yukari Viewpoint Survey, which was administered to anatomy students after the completion of the course. It was designed to evaluate students’ impressions and satisfaction with their team and the assignment method.

**Figure S1.** Comparison of medical education in Japan and the United States up to residency. The medical education systems in Japan and the United States have been summarized and aligned in a diagram for comparison. Equivalent processes are indicated by bars of the same color. The vertical axis represents the number of years post‐high school graduation. NEMP = National Examination for Medical Practitioners (Japan). CATCC = Common Achievement Tests to Clinical Clerkship, followed by clinical clerkship at hospitals (Japan). USMLE = US Medical Licensing Examination. MCAT = Medical College Admission Test (USA). * Residency duration varies depending on the program.

**Figure S2.** Weekly timeline integrating the Yukari method with the gross anatomy course. A schematic weekly timeline illustrating incorporation of the Yukari method (green) into the gross anatomy course (purple). The diagram shows the sequence of key events, including the information session, peer preference survey, team assignment, anatomy lectures, dissection laboratories, surveys, and exams, highlighting integration of team formation with the anatomy course.

**Figure S3.** Computational cost of brute‐force and local search team assignment optimization. Computational costs of team assignment optimization were compared between brute‐force (red) and local search (blue). The evaluation assumes teams of 4 members each. The shaded regions indicate the computational cost by El Capitan, one of the fastest supercomputers as of June 2025, with a peak speed at 1.742 *EFlop/s*. The x‐axis shows class size (number of teams), and the y‐axis shows computational cost (log scale, base 10).

**Figure S4.** Trends of students’ satisfaction with teams over the COVID‐19 pandemic. Students’ satisfaction on a Likert scale (1–5; higher is better) from 2012 to 2022 is summarized as a Likert plot. Note that high levels of student satisfaction were maintained over the COVID‐19 pandemic.

**Table S1.** Scoring of examinations and assignments. This table provides an example of the point allocation for examinations and assignments for the Class of 2013, along with the corresponding average scores and standard deviations.

**Table S2.** Summary of surveys. This table summarizes the surveys conducted each year in this study, detailing the class year, type of survey, assignment method, survey mode, survey date, number of participants and response rate.

**Table S3.** Summary of statistical methods. This table summarizes the statistical methods used in the study including data type, nature of data, number of cohorts, and the statistical test used for each figure or table.
