# Supplementary material for: Optimizing anatomy dissection teams using the Yukari method: A peer compatibility‐based approach
Source: Anat Sci Educ. 2025 Oct 3;18(11):1262–77. doi: 10.1002/ase.70124 (PMC12592916; doi:10.1002/ase.70124)
Supplement: Supplementary file 1 — Appendix A1. Yukari peer preference survey 2. This appendix provides the full text of the peer preference survey (version 2), which was administered to all anatomy students via a secure Google Form. It was designed to collect ratings of students’ commitment to the dissection lab and their anticipated peer preferences for use in team optimization. [file ASE-18-1262-s003.docx]

# APPENDIX A1: Yukari Peer Preference Survey 2

The following survey is implemented on a secure Google Form (Google, Mountain View, CA) and is administered to all students prior to the start of the anatomy course for use in Yukari Method team optimization.

## Anatomy Team Preference Survey

Deadline: the first Monday of the coming August

Your anatomy class starts this September. You will participate in anatomy dissection in a team of four for three months, so your success in this course largely depends on the team assignment. We, the anatomy faculty, use a computer-optimized system to assign teams based on your personal preferences collected through this secure survey.

Given the sensitivity of the information, only one faculty member, bound by confidentiality, will handle the data. The collected information will be used exclusively for team assignments and will not be disclosed to any parties, even if an individual requests their own data. Your responses to this survey will not affect your academic scores. (†)

† Anonymized and/or statistical data may be used to improve the assignment program and for scientific reports.

* Required questions

### Will you respond this survey? *

By choosing “No”, the survey will end without your participation and your team assignment will be done using default options.

- Yes
- No

### How committed are you to studying anatomy dissection?

Please let us know about your level of commitment to anatomy. Be honest—don't exaggerate or downplay your dedication. Choose the single option that best describes you.

### Motivation for Anatomy Dissection *

How much do you agree with the following statement? – "I am looking forward to anatomy dissection and am willing to do my best."

- Agree
- Somewhat agree
- Neither agree nor disagree
- Somewhat disagree
- Disagree

### Time available for anatomy dissection *

Anatomy dissection can be time-consuming and may extend beyond the scheduled time, sometimes lasting until 8 PM when the dissection room closes. How late are you willing to work on dissection?

- I am willing to do dissection until late
- I am willing to do dissection until somewhat late
- Neither late nor early
- I am willing to finish dissection as scheduled
- I am willing to finish dissection early before scheduled time

### How do you evaluate your preference for each classmate?

Indicate how favorable it would be for you to be assigned to the same anatomy team as the classmate in question. Consider how much this teammate would enhance your academic performance, not necessarily how much you personally like the person.

5. Good

4. Somewhat good

3. Neither good nor bad

2. Somewhat bad

1. Bad

**Note:**An excessively biased response, such as choosing “5” for a few friends and “1” for all others, may make computer optimization difficult or even infeasible for the entire class. Please choose “1” or “2” for 10% or fewer of your classmates. If your selections of “1” or “2” exceed 10%, all of those responses will be replaced with “3.”

### Your peer preferences

Choose one option per line. If you do not make a selection, the default value will be set to "3".

| # | Name | 5 | 4 | 3 | 2 | 1 |
| --- | --- | --- | --- | --- | --- | --- |
| 16001 | Adachi, B | 🔾 | 🔾 | 🔾 | 🔾 | 🔾 |
| 16002 | Cajal, S | 🔾 | 🔾 | 🔾 | 🔾 | 🔾 |
| 16003 | Chubb, D | 🔾 | 🔾 | 🔾 | 🔾 | 🔾 |
| 16004 | Corti, AGG | 🔾 | 🔾 | 🔾 | 🔾 | 🔾 |
| 16005 | Gray, H | 🔾 | 🔾 | 🔾 | 🔾 | 🔾 |
| 16006 | Harvey, W | 🔾 | 🔾 | 🔾 | 🔾 | 🔾 |
| 16007 | Meckel, JF | 🔾 | 🔾 | 🔾 | 🔾 | 🔾 |
| ... | ... | 🔾 | 🔾 | 🔾 | 🔾 | 🔾 |

#### [SUBMIT BOTTON]
