# Supplementary material for: Optimizing anatomy dissection teams using the Yukari method: A peer compatibility‐based approach
Source: Anat Sci Educ. 2025 Oct 3;18(11):1262–77. doi: 10.1002/ase.70124 (PMC12592916; doi:10.1002/ase.70124)
Supplement: Supplementary file 2 — Appendix A2. The Yukari Code 2.0 and related files. This appendix provides the source code, executables, sample data, and supporting files of the Yukari Code 2.0 program, which implements the heuristic local search algorithm for team optimization. These files are included as Supplementary Materials and are available for replication or local implementation of the Yukari method. [file ASE-18-1262-s002.docx]

# Appendix A2: The Yukari Code

The Yukari Code, executables (for macOS and Windows), sample data, README files, and support tools for the local implementation of the Yukari Method are available under the MIT license in the GitHub repository: <https://github.com/tohru-murakami/Yukari_Code_2> (Murakami and Araki, 2024).

Although the detailed algorithm is provided in the source code, a brief outline of the optimization procedure is presented here in seven steps:

1. Create a tentative initial solution (assignment) by random assortment.
2. Select two teams at random, and then choose one student at random from each of the two teams.
3. Evaluate the amount of change in class score (average team compatibility) when the two students are traded.
4. Repeat this random pick‑and‑swap evaluation *np* times, selecting the pair that maximizes the class score. The value of *np* is set to 0.1**N*(*N*–1)/2, where *N* is the number of students in the class.
5. If the swap improves the class score, perform the trade and then return to Step 2. Exit the loop if none of the *np* pairs increases the class score or the repeat count exceeds *N_LOOP* = 1000. Add the assignment to the list of candidates of the best optimized assignment.
6. Restart the search from Step 1 to add another candidate assignment. Repeat this *TRIAL* = 10 times.
7. Return the candidate assignment with the highest class score.

This appendix include in the source code and supplementary materials for Yukari Code 2.0.

- **README.txt:** Explains how to use the code.
- **grouping_ils.cpp:** The source code.
- **grouping_ils.hpp**: The header
- **Makefile:** Allows you to build a binary executable simply by typing “make.”
- **make_100_team_assignments.sh**: Shell script for making 100 assignments.
- **data.txt:** sample data of 64 students.
- **results.txt**: sample output of 100 assignments

## File “README.txt”

Group Optimization Program

Time-stamp: <2018-05-22 16:00:18 arakit>

** Changes

The calculation has been modified to complete within the specified time.

The criteria for a good group division have been changed.

The number of transfer students in a group is no longer considered.

** The problem this program solves

The task is to create groups of m students from the class of n students. The "compatibility" between students is given as a number from 1 to MAX_COMPATIBILITY. A larger number indicates a "better relationship."

For example, the goal is to create groups that satisfy the following conditions:

(1) Each group has m or m-1 members.

(2) Groups with only one girl or one boy are not allowed, except in groups of three where one can be alone.

(3) A "good group assignment" is defined as:

(a) The group with the largest "minimum compatibility" within the group.

(b) If the "minimum compatibility" is the same, the group with the largest "total compatibility" is preferred.

** Assumptions

(1) There are more boys than girls.

(2) If girls cannot be alone in a group, the number of girls is even. (If the number of girls is odd, please add a dummy student.)

** Preparing Student Data

Prepare a file as follows:

Example:

--- Start ---

16

0 1 0

1 1 0

2 1 0

3 1 0

4 1 0

5 1 1

6 0 1

7 0 1

8 0 0

9 0 0

10 0 0

11 0 0

12 0 0

13 0 0

14 0 0

15 0 0

0 1 4

0 2 3

0 3 9

0 4 3

0 5 6

0 6 2

0 7 2

0 8 5

0 9 7

0 10 6

0 11 1

0 12 3

0 13 7

0 14 7

0 15 6

1 2 4

1 3 6

1 4 6

1 5 4

1 6 7

1 7 6

1 8 5

1 9 9

1 10 7

1 11 3

1 12 5

1 13 4

1 14 5

1 15 5

2 3 8

2 4 6

2 5 5

2 6 3

2 7 8

2 8 3

2 9 5

2 10 5

2 11 4

2 12 6

2 13 3

2 14 5

2 15 7

3 4 9

3 5 9

3 6 6

3 7 6

3 8 4

3 9 4

3 10 7

3 11 6

3 12 10

3 13 4

3 14 6

3 15 5

4 5 1

4 6 9

4 7 5

4 8 7

4 9 10

4 10 7

4 11 7

4 12 2

4 13 4

4 14 6

4 15 8

5 6 8

5 7 8

5 8 4

5 9 8

5 10 4

5 11 4

5 12 5

5 13 7

5 14 5

5 15 8

6 7 4

6 8 4

6 9 8

6 10 4

6 11 6

6 12 5

6 13 6

6 14 5

6 15 9

7 8 5

7 9 9

7 10 7

7 11 4

7 12 4

7 13 6

7 14 9

7 15 5

8 9 5

8 10 3

8 11 6

8 12 2

8 13 3

8 14 6

8 15 4

9 10 5

9 11 4

9 12 6

9 13 4

9 14 4

9 15 5

10 11 5

10 12 2

10 13 9

10 14 8

10 15 5

11 12 8

11 13 4

11 14 3

11 15 9

12 13 5

12 14 9

12 15 2

13 14 4

13 15 5

14 15 6

0 0 0

--- End ---

* File Description

Line 1: Total number of students n

Next n lines:

Student ID, Gender, Transfer student status

Gender (0: Male, 1: Female)

Transfer student (0: Regular, 1: Transfer)

Subsequent lines:

Format: a b x where a and b are students, and x indicates their relationship.

The last line ends with "0 0 0".

** Pre-configuration of the program

In the file grouping_ils.cpp, there is a section titled "Configurable Parameters." Adjust the following variables according to your objectives:

(1) MAX_COMPATIBILITY: The maximum value for compatibility. In the following example, it is 10:

const int MAX_FRIENDSHIP = 10;

(2) STUDENT_IN_GROUP: The number of students per group. If the total number is not divisible, groups with one less member are created. In this example, groups of 4 or 3 members are created:

const int STUDENT_IN_GROUP = 4;

(3) PermitGirlAlone: Set to true if you allow groups with only one girl, false otherwise. In the following example, groups with only one girl are not allowed:

const bool PermitGirlAlone = false;

(4) PermitBoyAlone: Set to true if you allow groups with only one boy, false otherwise. In the following example, groups with only one boy are not allowed:

const bool PermitBoyAlone = false;

(5) PermitAllGirl: Set to true to allow all-girl groups:

const bool PermitAllGirl = false;

(6) PermitAllBoy: Set to true to allow all-boy groups:

const bool PermitAllBoy = true;

(Note) Variables set with true or false might not function correctly as they have not been thoroughly tested. Please contact us if you notice any issues.

(7) LIMIT_TIME: The time (in seconds) allowed for searching for a solution. The loop terminates if the time exceeds this value. In the following example, it stops after 120 seconds:

double LIMIT_TIME = 120;

** Compilation

Use the make command.

Example:

% make

This generates the executable file grouping_ils. Run it as follows:

% ./grouping_ils filename

To specify the execution time, use -t time (seconds). If no time is specified, the LIMIT_TIME defined in the source file is used.

In the following example, the calculation stops after 10 seconds and outputs the result:

Example: If the file name in the above example is `data16.txt`:

% ./grouping_ils -t 10 data16.txt

Output Example:

=======

0,4,7H,10,13,14,7 6 9 9 8 4 ,41.0,43,40,4,1,

1,4,1g,4g,6H,9,6 7 9 9 10 8 ,49.0,49,62,2,1,

2,4,0g,5gH,8,15,6 5 6 4 8 4 ,29.0,33,42,2,1,

3,4,2g,3g,11,12,8 4 6 6 10 8 ,40.0,42,42,2,0,

Total Value = 159.0

Minimum Compatibility = 4

Total Compatibility = 167

=======

From left to right:

Group number

Number of members in the group (m)

Student numbers of the m members. g indicates a girl, H indicates a transfer student.

List of compatibilities within the group

Group's evaluation value (reduced by the lowest compatibility)

Total compatibility within the group

Minimum compatibility within the group

Number of girls

Number of boys

Number of transfer students

## File “grouping_ils.cpp”

#include <iostream>

#include <fstream>

#include <cstdio>

#include <cstdlib>

#include <random>

#include <vector>

#include <algorithm>

#include <tuple>

#include <ctime>

#include "grouping_ils.hpp"

#include <unistd.h>

using namespace std;

// If this is set to true, various output will be generated.

//bool DEBUG = true;

bool DEBUG = false;

/*****************************************************

*

* Configurable parameters

*

*****************************************************/

// (1) Maximum value of peer compatibility

const int MAX_COMPATIBILITY = 10;

// (2) Number of people in one group (dissection team)

// If the class is not divisible by the number, groups of 1 less people is allowed.

const int STUDENTS_IN_GROUP = 4;

// (3) True when groups with single girl are allowed.

// One girl in a group of 3 or less is allowed.

const bool PermitGirlAlone = false;

// (4) True when groups with single boy are allowed.

// One boy in a group of 3 or less is allowed.

const bool PermitBoyAlone = false;

// (5) True if groups of all girls are allowed.

const bool PermitAllGirl = true;

// (6) True if groups of all boys are allowed.

const bool PermitAllBoy = true;

// (7) True if more than one transfer student is allowed in a group.

//const bool PermitTwoHennyu = false;

// (7) default value of maximum processing time in seconds.

double LIMIT_TIME = 300;

/*** End: Configurable parameters *********************/

// Penalty value of peer compatibility

double factor = 2.0;

// Number of repeats for 3rd depth neighbor search

// If this is set to 0, no 3rd depth neighbors will be searched.

int try3swap = 1;

// If true, the first found 3rd depth better neighbor will be selected.

// When false, the best neighbor will be selected.

bool first_find_in_3swap = true;

/*************************************************/

// Global variable (no configuration needed)

// the maximum number of students

const int MAX_STUDENTS = 150;

// the maximum number of groups

const int MAX_NGROUP = 35;

// the maximum number of persons of groups

const int MAX_SIZE = 6;

const int DUMMY = 10000;

// Array to store peer compatibility

int COMPATIBILITY[MAX_STUDENTS][MAX_STUDENTS];

// Array to store group assignment

int GROUPS[MAX_NGROUP][MAX_SIZE];

// Number of groups

int numGroups = 0;

// Number of students

int numStudents = 0;

// The minimum value of peer compatibilities in a group

int minimumCompatibility;

// Number of peers with the minimum compatibility value

int numMinCompatibility;

// Depth to the optimum solution

int inline default_distance() { return numGroups; }

// Number of feasible solutions

int NUMFEASIBLESOLUTION = 0;

/*************************************************/

Student STUDENTS[MAX_STUDENTS];

/*************************************************/

// Manage group information

GI groupInfo[MAX_NGROUP];

int best_grouping[MAX_NGROUP][MAX_SIZE];

int best_size[MAX_NGROUP];

// Set to true when any feasible solution is found．

bool FoundFeasibleSolution = false;

// seed of random number

unsigned int Seed;

/*************************************************/

typedef struct edge

{

int group;

int x;

int y;

} Edge;

typedef struct gs

{

int group;

int id;

} GS;

/** Auxiliary functions *******************************************************/

// Generate random swaps within {0,...,n-1} and return as a vector.

vector<int> random_permutation(int n, CRand& mt)

{

vector<int> rv;

for (int i = 0; i < n; i++)

{

rv.push_back(i);

}

for (int i = 0; i < n-1; i++)

{

//auto num = mt.get();

int num = mt.get();

int j = (num % n-i) + i;

int tmp = rv[i];

rv[i] = rv[j];

rv[j] = tmp;

}

return rv;

}

/** end: Auxiliary functions **************************************************/

/*** Read data from a file ***/

int read_file(const string &filename, Student *STUDENTS, int COMPATIBILITY[MAX_STUDENTS][MAX_STUDENTS])

{

ifstream ifs(filename.c_str());

int count_friendship[MAX_COMPATIBILITY + 1];

if (ifs.fail())

{

cerr << "File does not exist.\n";

exit(1);

}

ifs >> numStudents; // Total number of students

for (int i = 0; i < numStudents; i++)

{

string sn; // student number

int gn, hn; // gender, transfer

ifs >> sn >> gn >> hn;

Student st;

st.id = i;

st.studentNumber = sn;

st.gender = gn;

st.hennyu = hn;

STUDENTS[i] = st;

}

// store the friendship level

int p1, p2, v;

for (int i = 0; i < numStudents; i++) COMPATIBILITY[i][i] = 0;

while (ifs >> p1 >> p2 >> v)

{

if ((p1==0) && (p2==0) && (v==0)) return 0;

if ((v <= 0) || (v > MAX_COMPATIBILITY))

{

cerr << "Irregal value of friendship" << endl;

exit(1);

}

COMPATIBILITY[p1][p2] = COMPATIBILITY[p2][p1] = v;

count_friendship[v]++;

}

return 1;

}

/*** End: read_file Read data from a file ***/

/*** Add student i to group k ***/

int joinStudent(int GROUPS[][MAX_SIZE], int k, int i)

{

int j = 0;

while (GROUPS[k][j] < numStudents)

{

j++;

}

GROUPS[k][j] = i;

groupInfo[k].size++;

return k;

}

/*** remove student i from group k

return 1 when the student exists, else 0

***/

int removeStudent(int k, int i)

{

int j;

bool found = false;

if (groupInfo[k].size == 0) return 0;

for (j = 0; j < groupInfo[k].size; j++)

{

if (i == GROUPS[k][j])

{

found = true;

break;

}

}

if (found)

{

GROUPS[k][j] = DUMMY;

groupInfo[k].size--;

sort(GROUPS[k], GROUPS[k] + MAX_SIZE);

return 1;

}

else

return 0;

}

/*** swap student s1 in group k1 with s2 in k2 ***/

/*** return 1 when successful, else 0 ***/

int swap_students(int k1, int s1, int k2, int s2)

{

if (k1 == k2) return 0;

int s1_sucess = removeStudent(k1, s1);

int s2_sucess = removeStudent(k2, s2);

if (!s1_sucess && !s2_sucess) return 0;

else if (!s1_sucess && s2_sucess)

{

joinStudent(GROUPS, k2, s2);

return 0;

}

else if (s1_sucess && !s2_sucess)

{

joinStudent(GROUPS, k1, s1);

return 0;

}

else

{

joinStudent(GROUPS, k1, s2);

joinStudent(GROUPS, k2, s1);

return 1;

}

}

/*** move student s1 in group k1 to group k2 ***/

/*** return 1 when successful, else 0 ***/

int move_student(int k1, int s1, int k2)

{

if (k1 == k2) return 0;

int s1_sucess = removeStudent(k1, s1);

if (!s1_sucess) return 0;

else

{

joinStudent(GROUPS, k2, s1);

return 1;

}

}

/*** make_initial_group : generate groups by random assortment ***/

void make_initial_group(CRand &mt)

{

// number of groups

numGroups = numStudents / STUDENTS_IN_GROUP;

if (numStudents % STUDENTS_IN_GROUP > 0) numGroups++;

// initialize

for (int k=0; k<numGroups; k++)

{

for (int i=0; i<MAX_SIZE; i++)

{

GROUPS[k][i] = DUMMY;

}

}

// calculate number of students per group

for (int k=0; k<numGroups; k++)

{

groupInfo[k].size = 0;

}

vector<int> rv = random_permutation(numStudents, mt);

int s = 0;

int k = 0;

for (s=0; s < numStudents; s++)

{

joinStudent(GROUPS, k, rv[s]);

if (k == numGroups - 1) k = 0;

else k++;

}

} // End: make_initial_group

/*** Display list of group k ***/

void printGroupCsv(int k)

{

int* vs = GROUPS[k];

sort(vs, vs + groupInfo[k].size);

// Group number and size

printf("%d,%d,", k, groupInfo[k].size);

// Member list

for (int i = 0; i < groupInfo[k].size; i++)

{

int st = vs[i];

printf("%d", st);

if (STUDENTS[st].gender && STUDENTS[st].hennyu) printf("gH,");

else if (STUDENTS[st].gender && !STUDENTS[st].hennyu) printf("g,");

else if (!STUDENTS[st].gender && STUDENTS[st].hennyu) printf("H,");

else printf(",");

}

// Insert a blank space for groups with one less member

if (groupInfo[k].size < STUDENTS_IN_GROUP)

{

printf(" ,");

}

// Compatibility list within the group

vector<int> edges;

for (int i = 0; i < groupInfo[k].size; i++)

{

for (int j = i + 1; j < groupInfo[k].size; j++)

{

int s1 = GROUPS[k][i];

int s2 = GROUPS[k][j];

int r = COMPATIBILITY[s1][s2];

edges.push_back(r);

}

}

for (int i=0; i < (int)edges.size(); i++)

{

printf("%d ", edges[i]);

}

printf(",");

printf("%.1f,%d,", groupInfo[k].evaluation, groupInfo[k].totalCompatibility);

printf("%d,%d,%d,", groupInfo[k].numGirls, groupInfo[k].numBoys, groupInfo[k].numH);

// Display NG for groups that violate the rules

if (!groupInfo[k].feasible) printf("NG\n");

else printf("\n");

}

/*** End : PrintGroupCsv ***/

/*** Print list of groups ***/

void printAllGroup()

{

double tv = 0.0;

int tc = 0;

for (int k = 0; k < numGroups; k++)

{

//printGroup(k);

printGroupCsv(k);

tv += groupInfo[k].evaluation;

tc += groupInfo[k].totalCompatibility;

}

printf("Total Value = %3.1f\n", tv);

printf("Minimum Compatibility = %d\n", minimumCompatibility);

//printf("Num of Minimum Compatibility = %d\n", numMinCompatibility);

printf("Total Compatibility = %d\n", tc);

}

/*** End : PrintAllgroup ***/

/***

* gr[]: Array of students, gsize: Number of elements in the array

* Returns the evaluation value of the group

***/

double calc_group_evaluation(int *gr, int gsize)

{

double val = 0.0; // Evaluation value of the group

for (int i = 0; i < gsize; i++)

{

for (int j = i + 1; j < gsize; j++)

{

int s1 = gr[i];

int s2 = gr[j];

val += calc_evaluation(s1, s2);

}

}

// Lower the evaluation value of the group as a penalty if it violates constraints

int ng = 0;

int nb = 0;

int nh = 0;

for (int i = 0; i < gsize; i++)

{

Student s = STUDENTS[gr[i]];

if (s.gender) ng++; else nb++;

if (s.hennyu) nh++;

}

// Increase the penalty for violations of the constraints

int penalty = 0;

if (!PermitGirlAlone && (ng == 1) && (gsize >=4)) penalty++;

if (!PermitBoyAlone && (nb == 1) && (gsize >=4)) penalty++;

if (!PermitAllGirl && (nb == 0)) penalty++;

if (!PermitAllBoy && (ng == 0)) penalty++;

//if (!PermitTwoHennyu && (nh > 1)) penalty++;

// Decrease the evaluation value by the penalty

double eval;

if (val >= 0) eval = val / (1 + penalty); // <-- Reduction of evaluation

else eval = val * (1 + penalty); // <-- Reduction of evaluation

return eval;

}

/*********************************************************************/

// Update group information in groupInfo[MAX_NGROUP]

void update_groupInfo(int k)

{

double val = 0.0; // Evaluation value of the group

int mc = MAX_COMPATIBILITY; // Minimum compatibility

int num_mc = 0; // Number of pairs with minimum compatibility

int tc = 0; // Total compatibility

int sz = 0; // Number of people

for (int i = 0; i < MAX_SIZE; i++)

if (GROUPS[k][i] < MAX_STUDENTS) sz++;

groupInfo[k].size = sz;

for (int i = 0; i < sz; i++)

{

for (int j = i + 1; j < sz; j++)

{

int s1 = GROUPS[k][i];

int s2 = GROUPS[k][j];

int f = COMPATIBILITY[s1][s2];

if (f < mc)

{

mc = f;

}

tc += f;

val += calc_evaluation(s1, s2);

}

}

for (int i = 0; i < sz; i++)

for (int j = i + 1; j < sz; j++)

{

int s1 = GROUPS[k][i];

int s2 = GROUPS[k][j];

int f = COMPATIBILITY[s1][s2];

if (f == mc) num_mc++;

}

groupInfo[k].minCompatibility = mc;

groupInfo[k].numMinCompatibility = num_mc;

for (int i = 0; i < sz; i++)

for (int j = i + 1; j < sz; j++)

{

int s1 = GROUPS[k][i];

int s2 = GROUPS[k][j];

if (s1 == s2)

{

printf("Student %d is doubled in Group %d\n", s1, k);

printf("Seed = %u\n", Seed);

exit(1);

}

}

// Validate students

int g = 0, b = 0, h = 0;

for (int i = 0; i < sz; i++)

{

int s = GROUPS[k][i];

Student st = STUDENTS[s];

if (st.gender) g++; else b++;

if (st.hennyu) h++;

}

groupInfo[k].numGirls = g;

groupInfo[k].numBoys = b;

groupInfo[k].numH = h;

// Lower the evaluation value of the group as a penalty if it violates constraints

// Increase the penalty for violations of the constraints

int penalty = 0;

bool feasible = true;

if (!PermitGirlAlone && (groupInfo[k].numGirls == 1) && (groupInfo[k].size >= 4))

{

penalty++;

feasible = false;

}

if (!PermitBoyAlone && (groupInfo[k].numBoys == 1) && (groupInfo[k].size >= 4))

{

penalty++;

feasible = false;

}

if (!PermitAllGirl && (groupInfo[k].numBoys == 0)) { penalty++; feasible = false; }

if (!PermitAllBoy && (groupInfo[k].numGirls == 0)) { penalty++; feasible = false; }

//if (!PermitTwoHennyu && (groupInfo[k].numH > 1)) { penalty++; feasible = false; }

// Decrease the evaluation value by the penalty

if (val >= 0) groupInfo[k].evaluation = val / (1 + penalty); // <-- Reduction of evaluation

else groupInfo[k].evaluation = val * (1 + penalty);

groupInfo[k].totalCompatibility = tc;

groupInfo[k].numGirls = g;

groupInfo[k].numBoys = b;

groupInfo[k].numH = h;

groupInfo[k].feasible = feasible;

}

// End: update_groupInfo

// Return the difference in evaluation values after swapping student s1 from group k1 with student s2 from group k2

// Positive return value indicates an increase in evaluation value after the swap

double delta_by_swap(int k1, int s1, int k2, int s2)

{

// Evaluation value of the groups before the swap

double before = groupInfo[k1].evaluation + groupInfo[k2].evaluation;

// Generate groups after the swap

int group_k1[MAX_SIZE];

int group_k2[MAX_SIZE];

int j = 0;

for (int i=0; i < groupInfo[k1].size; i++)

{

int s = GROUPS[k1][i];

if (s != s1)

{

group_k1[j] = s;

j++;

}

}

group_k1[groupInfo[k1].size - 1] = s2;

j = 0;

for (int i=0; i < groupInfo[k2].size; i++)

{

int s = GROUPS[k2][i];

if (s != s2)

{

group_k2[j] = s;

j++;

}

}

group_k2[groupInfo[k2].size - 1] = s1;

// Evaluation value after the swap

double after = calc_group_evaluation(group_k1, groupInfo[k1].size) +

calc_group_evaluation(group_k2, groupInfo[k2].size);

// Return the difference in evaluation values

return after - before;

} // end delta_by_swap

// Return the difference in evaluation values after moving student s1 from group k1 to group k2

// Positive return value indicates an increase in evaluation value after the move

double delta_by_move(int k1, int s1, int k2)

{

// Evaluation value of the groups before the move

double before = groupInfo[k1].evaluation + groupInfo[k2].evaluation;

// Generate groups after the move

int group_k1[MAX_SIZE];

int group_k2[MAX_SIZE];

int j = 0;

for (int i=0; i < groupInfo[k1].size; i++)

{

int s = GROUPS[k1][i];

if (s != s1)

{

group_k1[j] = s;

j++;

}

}

for (int i=0; i < groupInfo[k2].size; i++)

{

int s = GROUPS[k2][i];

group_k2[i] = s;

}

group_k2[groupInfo[k2].size] = s1;

// Evaluation value after the move

double after = calc_group_evaluation(group_k1, groupInfo[k1].size - 1) +

calc_group_evaluation(group_k2, groupInfo[k2].size + 1);

// Return the difference in evaluation values

return after - before;

} // end delta_by_move

// Return the difference in evaluation values after swapping (k1, s1) -> (k2, s2) -> (k3, s3)

// Positive return value indicates an increase in evaluation value after the swap

double delta_by_3swap(int k1, int s1, int k2, int s2, int k3, int s3)

{

// Evaluation value of the groups before the swap

double before = groupInfo[k1].evaluation + groupInfo[k2].evaluation + groupInfo[k3].evaluation;

// Generate groups after the swap

int group_k1[STUDENTS_IN_GROUP];

int group_k2[STUDENTS_IN_GROUP];

int group_k3[STUDENTS_IN_GROUP];

// s3 -> k1

int j = 0;

for (int i=0; i < groupInfo[k1].size; i++)

{

int s = GROUPS[k1][i];

if (s != s1)

{

group_k1[j] = s;

j++;

}

}

group_k1[groupInfo[k1].size - 1] = s3;

// s1 -> k2

j = 0;

for (int i=0; i < groupInfo[k2].size; i++)

{

int s = GROUPS[k2][i];

if (s != s2)

{

group_k2[j] = s;

j++;

}

}

group_k2[groupInfo[k2].size - 1] = s1;

// s2 -> k3

j = 0;

for (int i=0; i < groupInfo[k3].size; i++)

{

int s = GROUPS[k3][i];

if (s != s3)

{

group_k3[j] = s;

j++;

}

}

group_k3[groupInfo[k3].size - 1] = s2;

// Evaluation value after the swap

double after = calc_group_evaluation(group_k1, groupInfo[k1].size)

+ calc_group_evaluation(group_k2, groupInfo[k2].size)

+ calc_group_evaluation(group_k3, groupInfo[k3].size);

// Return the difference in evaluation values

return after - before;

} // end calc_eval_by_3swap

/*** Simple local search ***/

int local_search_swap(CRand &mt)

{

// Return true if a local optimum solution is found

bool found_local_opt = false;

do

{

// Evaluate all neighboring swaps

vector<pair<GS,GS> > exchange_pair;

double max_difference = 0.0;

int k1, k2;

int p1, p2;

for (k1 = 0; k1 < numGroups; k1++)

{

for (k2 = k1+1; k2 < numGroups; k2++)

{

if (groupInfo[k1].size == groupInfo[k2].size)

{

// Check 2-swap neighbors as the group sizes are the same

for (p1 = 0; p1 < groupInfo[k1].size; p1++)

{

for (p2 = 0; p2 < groupInfo[k2].size; p2++)

{

int s1 = GROUPS[k1][p1];

int s2 = GROUPS[k2][p2];

// Difference in evaluation value after the swap

double delta = delta_by_swap(k1, s1, k2, s2);

GS gs1, gs2;

if (delta >= max_difference && delta > 0)

{

if (delta > max_difference)

{

max_difference = delta;

exchange_pair.clear();

}

gs1.group = k1;

gs1.id = s1;

gs2.group = k2;

gs2.id = s2;

exchange_pair.push_back(make_pair(gs1, gs2));

}

}

}

} // end 2-swap neighbors (groups k1 and k2 have the same size)

else

{

// Check 1-move neighbors

if (groupInfo[k1].size - groupInfo[k2].size == 1)

{

// Move a student from k1 to k2

for (int p = 0; p < groupInfo[k1].size; p++)

{

int s1 = GROUPS[k1][p];

// Difference in evaluation value after the move

double delta = delta_by_move(k1, s1, k2);

GS gs1, gs2;

if (delta >= max_difference && delta > 0)

{

if (delta > max_difference)

{

max_difference = delta;

exchange_pair.clear();

}

gs1.group = k1;

gs1.id = s1;

gs2.group = k2;

gs2.id = -1;

exchange_pair.push_back(make_pair(gs1, gs2));

}

}

}

else

{

// Move a student from k2 to k1

for (int p = 0; p < groupInfo[k2].size; p++)

{

int s2 = GROUPS[k2][p];

// Difference in evaluation value after the move

double delta = delta_by_move(k2, s2, k1);

GS gs1, gs2;

if (delta >= max_difference && delta > 0)

{

if (delta > max_difference)

{

max_difference = delta;

exchange_pair.clear();

}

gs1.group = k2;

gs1.id = s2;

gs2.group = k1;

gs2.id = -1;

exchange_pair.push_back(make_pair(gs1, gs2));

}

} // for p

} // end else

} // end 1-move neighbors check

} // end for k2

} // Check all neighbors

// exchange_pair contains the pair of neighbors with the highest evaluation

// If the second element is -1, it means moving a student

if (exchange_pair.empty())

{

found_local_opt = true;

if (DEBUG)

{

printf("------------\n");

printf(" Found Local Optima by 2-swap.\n");

}

}

else

{

int p = mt.get() % exchange_pair.size();

GS gs1 = exchange_pair[p].first;

GS gs2 = exchange_pair[p].second;

k1 = gs1.group;

k2 = gs2.group;

int s1 = gs1.id, s2 = gs2.id;

if (DEBUG)

{

if (s2 == -1)

printf(" Move (%d,%d) --> %d; %.1f\n", k1, s1, k2, max_difference);

else

printf(" Swap (%d,%d) <--> (%d,%d); %.1f\n", k1, s1, k2, s2, max_difference);

}

if (s2 == -1)

{

// Move student

if (DEBUG)

{

// Check if s1 is already in group k2

for (int j=0; j<groupInfo[k2].size; j++)

{

if (s1 == GROUPS[k2][j])

{

printf("Student %d is already in Group %d\n", s1, k2);

exit(1);

}

}

}

move_student(k1, s1, k2);

}

else

{

// Swap students (k1,s1) <==> (k2,s2)

if (DEBUG)

{

// Check if s1 is already in group k2

for (int j=0; j<groupInfo[k2].size; j++)

{

if (s1 == GROUPS[k2][j])

{

printf("Student %d is already in Group %d\n", s1, k2);

exit(1);

}

}

// Check if s2 is already in group k1

for (int j=0; j<groupInfo[k1].size; j++)

{

if (s2 == GROUPS[k1][j])

{

printf("Student %d is already in Group %d\n", s2, k1);

exit(1);

}

}

}

// Swap s1 and s2

swap_students(k1, s1, k2, s2);

} // End of student exchange

update_groupInfo(k1);

update_groupInfo(k2);

//if (DEBUG) printAllGroup();

}

} while (!found_local_opt);

return 1;

} /* End: local_search */

/*** Using 3-swap to find a better solution in the neighborhood

*** Try 3-swap neighborhood for try3swap times

*** Move to the first improvement found if first_find == true

*** Move to the best improvement found if first_find == false

*** Return 1 if a better neighborhood is found

*** Return 0 otherwise ***/

int local_search_by_3swap(int try3swap, bool first_find, CRand &mt)

{

if (try3swap == 0) return 0;

if (DEBUG) printf(" Try 3-swap\n");

double max_difference = 0.0;

int k1, k2, k3;

int p1, p2, p3;

vector<tuple<GS, GS, GS> > exchange_triple;

// Number of 3-Swap iterations

int IterationTimeof3Swap = 0;

while (IterationTimeof3Swap < try3swap)

{

IterationTimeof3Swap++;

// Number of 3-Swap iterations

k1 = mt.get() % numGroups;

do {

k2 = mt.get() % numGroups;

} while (k1 == k2);

do {

k3 = mt.get() % numGroups;

} while ((k3 == k1) || (k3 == k2));

// Selected groups k1, k2, k3

for (p1 = 0; p1 < groupInfo[k1].size; p1++)

{

for (p2 = 0; p2 < groupInfo[k2].size; p2++)

{

for (p3 = 0; p3 < groupInfo[k3].size; p3++)

{

int s1 = GROUPS[k1][p1];

int s2 = GROUPS[k2][p2];

int s3 = GROUPS[k3][p3];

// Calculate the difference in evaluation value after the swap

double delta1 = delta_by_3swap(k1, s1, k2, s2, k3, s3);

double delta2 = delta_by_3swap(k3, s3, k2, s2, k1, s1);

double delta = max(delta1, delta2);

if (first_find)

{

// if first_find == true

if (delta > 0)

{

if (delta1 > delta2)

{

// s1 -> s2 -> s3

swap_students(k1, s1, k2, s2);

swap_students(k2, s2, k3, s3);

}

else

{

// s1 -> s3 -> s2

swap_students(k1, s1, k2, s2);

swap_students(k1, s1, k3, s3);

}

// Found an improved solution, so end

return 1;

}

}

else

{

if (delta > 0)

{

if (delta >= max_difference)

{

if (delta > max_difference)

{

max_difference = delta;

exchange_triple.empty();

}

GS gs1, gs2, gs3;

gs1.group = k1;

gs1.id = s1;

gs2.group = k2;

gs2.id = s2;

gs3.group = k3;

gs3.id = s3;

if (delta1 > delta2)

{

exchange_triple.push_back(make_tuple(gs1, gs2, gs3));

}

else

{

exchange_triple.push_back(make_tuple(gs3, gs2, gs1));

}

}

}

}

}

}

}

} // End of search by 3-Swap

// If first_find is 0, terminate here (no improvement found)

if (first_find) return 0;

// exchange_tuple contains the 3-tuple with the highest evaluation value

if (!exchange_triple.empty())

{

// Randomly select one and swap

int p = mt.get() % exchange_triple.size();

GS gs1 = get<0>(exchange_triple[p]);

GS gs2 = get<1>(exchange_triple[p]);

GS gs3 = get<2>(exchange_triple[p]);

int k1 = gs1.group, s1 = gs1.id;

int k2 = gs2.group, s2 = gs2.id;

int k3 = gs3.group, s3 = gs3.id;

if (DEBUG)

{

printf("------------\n");

printf(" 3-Swap (%d,%d) -> (%d,%d) -> (%d,%d)\n", k1, s1, k2, s2, k3, s3);

}

swap_students(k1, s1, k2, s2);

swap_students(k2, s2, k3, s3);

return 1;

}

else

{

// No improvement found

if (DEBUG)

{

printf("------------\n");

printf(" Found Local Optima by 3-swap\n");

}

return 0;

} // End of 3-Swap

} // End: local_search_by_3swap

/*********************************************************************/

/*********************************************************************/

/*********************************************************************/

/*********************************************************************/

/*** Calculate evaluation value from compatibility between s1 and s2 ***/

double calc_evaluation(int s1, int s2)

{

if (s1 == s2) return 0;

int f = COMPATIBILITY[s1][s2];

int d = max(0, minimumCompatibility - f + 1);

f = f - factor * d;

return f;

}

/*********************************************************************/

// Perform random 2-swap times times

void random_swap(int times, CRand &mt)

{

if (DEBUG) printf(" random_swap: %d\n", times);

for (int p = 0; p < times; p++)

{

// Randomly select 2 groups

int k1 = mt.get() % numGroups, k2;

do {

k2 = mt.get() % numGroups;

} while (k1 == k2);

int p1 = mt.get() % groupInfo[k1].size;

int p2 = mt.get() % groupInfo[k2].size;

int s1 = GROUPS[k1][p1];

int s2 = GROUPS[k2][p2];

int SWAP_SUCESS = swap_students(k1, s1, k2, s2);

if (!SWAP_SUCESS)

{

printf("Error\n");

exit(1);

}

}

}

/*** Iterated Local Search ***/

double iterated_local_search(int best_grouping[][MAX_SIZE], int best_size[], CRand &mt, int try3swap, bool first_find)

{

int maximumTotalCompatibility = 0;

double maximumTotalValue = 0.0;

double previous_tc = -100.0;

clock_t iteration_start = clock(); // Start time

clock_t iteration_end = iteration_start; // End time

bool initialize = true;

/*** Termination condition ***/

// Terminate if time exceeds LIMIT_TIME seconds

while ((double)(iteration_end - iteration_start) / CLOCKS_PER_SEC < LIMIT_TIME)

{

if (initialize)

{

make_initial_group(mt);

initialize = false;

for (int k = 0; k < numGroups; k++) update_groupInfo(k);

minimumCompatibility = MAX_COMPATIBILITY;

numMinCompatibility = 0;

for (int k = 0; k < numGroups; k++)

if (groupInfo[k].minCompatibility < minimumCompatibility)

{

minimumCompatibility = groupInfo[k].minCompatibility;

}

for (int k = 0; k < numGroups; k++)

{

if (groupInfo[k].minCompatibility == minimumCompatibility)

{

numMinCompatibility += groupInfo[k].numMinCompatibility;

}

}

if (DEBUG)

{

printf("Initial Grouping\n");

for (int k = 0; k < numGroups; k++) update_groupInfo(k);

printAllGroup();

}

}

// Initialization of groups and calculation of minimumCompatibility and numMinCompatibility completed

// Start of local search

for (int k = 0; k < numGroups; k++) update_groupInfo(k);

int not_local_opt = 1;

do

{

// Move to local optimal solution

local_search_swap(mt);

// Try 3-swap

not_local_opt = local_search_by_3swap(try3swap, first_find_in_3swap, mt);

if (DEBUG)

{

if (not_local_opt) printf("Found better 3-neighbor\n");

for (int k = 0; k < numGroups; k++) update_groupInfo(k);

//printAllGroup();

}

} while (not_local_opt);

// Check current solution

int current_mc = MAX_COMPATIBILITY;

int current_tc = 0;

double current_tv = 0.0;

// Check minimum compatibility and total compatibility of current grouping

for (int k = 0; k < numGroups; k++)

{

update_groupInfo(k);

current_mc = min(current_mc, groupInfo[k].minCompatibility);

current_tc += groupInfo[k].totalCompatibility;

current_tv += groupInfo[k].evaluation;

}

// Check if the solution is feasible

bool feasible = true;

for (int k = 0; k < numGroups; k++)

{

if (!groupInfo[k].feasible)

{

feasible = false;

break;

}

}

// Update new solution as the best solution if it is feasible or no feasible solution has been found yet,

// and if it has a higher minimum compatibility or the same minimum compatibility but higher total compatibility

if ((feasible || !FoundFeasibleSolution)

&&

((minimumCompatibility < current_mc)

||

((minimumCompatibility == current_mc) && (maximumTotalCompatibility < current_tc)))

)

{

if (DEBUG) printAllGroup();

// Update as the best solution if it is feasible

if (feasible)

{

// Found one feasible solution

NUMFEASIBLESOLUTION++;

FoundFeasibleSolution = true;

} // if feasible

minimumCompatibility = current_mc;

maximumTotalCompatibility = current_tc;

maximumTotalValue = current_tv;

// Update best_grouping

for (int k=0; k<MAX_NGROUP; k++)

for (int i=0; i<MAX_SIZE; i++)

best_grouping[k][i] = DUMMY;

for (int k=0; k<numGroups; k++)

{

for (int i=0; i<groupInfo[k].size; i++)

{

best_grouping[k][i] = GROUPS[k][i];

}

best_size[k] = groupInfo[k].size;

}

} // // end if compatibility values are good

else

{

if (DEBUG)

{

if (!feasible)

printf(" Infeasible Solution\n");

else

printf(" Not Improved\n");

}

}

// Move to a neighborhood with distance step from the current solution

// If returning to the previous local solution, jump to a farther solution

int distance = default_distance();

if (current_tc == previous_tc)

{

distance += distance;

}

else

{

distance = default_distance();

previous_tc = current_tc;

}

random_swap(distance, mt);

// End time

iteration_end = clock();

}

return maximumTotalValue;

}

int main(int argc, char** argv)

{

string filename;

// int best_grouping[MAX_NGROUP][MAX_SIZE];

// int best_size[MAX_NGROUP];

random_device rd;

Seed = rd();

//Seed = 1066235738; // For debugging

CRand mt(Seed);

if (DEBUG) printf("Seed = %u\n", Seed);

int opt;

opterr = 0;

while ((opt = getopt(argc, argv, "t:")) != -1)

{

// Repeat until no more command line options

switch (opt) {

case 't':

LIMIT_TIME = atof(optarg);

break;

default:

// If unspecified option is passed

printf("Usage: %s [-t time] filename\n", argv[0]);

break;

}

}

if (argc <= 1)

{

cerr << "Please give a problem filename." << endl;

return 1;

}

else

{

filename = string(argv[optind]);

}

read_file(filename, STUDENTS, COMPATIBILITY);

cout << filename << endl;

// initialize

for (int k=0; k<MAX_NGROUP; k++)

{

for (int i=0; i<MAX_SIZE; i++)

{

best_grouping[k][i] = DUMMY;

}

}

// Iterated Local Search

iterated_local_search(best_grouping, best_size, mt, try3swap, first_find_in_3swap);

// Restore and display the optimal groups

for (int k=0; k<MAX_NGROUP; k++)

{

for (int i=0; i<MAX_SIZE; i++)

{

GROUPS[k][i] = DUMMY;

}

}

for (int k=0; k<numGroups; k++)

{

for (int i=0; i < best_size[k]; i++)

{

GROUPS[k][i] = best_grouping[k][i];

}

groupInfo[k].size = best_size[k];

}

for (int k=0; k<numGroups; k++) update_groupInfo(k);

printAllGroup();

//printf("%d feasible solutions are found.\n", NUMFEASIBLESOLUTION);

return 0;

}

## File “Grouping_ils.hpp”

#ifndef GR_H

#define GR_H

#include <vector>

using namespace std;

class CRand {

public:

// Constructor

CRand(int seed = 1)

: generator_(seed), distribution_(0, 65535) {}

~CRand(){}

// Getter

int get() { return distribution_(generator_); }

private:

std::mt19937 generator_;

std::uniform_int_distribution<> distribution_;

};

/*** Structure to handle group evaluation values, minimum compatibility, and total compatibility ***/

typedef struct gv

{

double eval;

int minCompatibility;

int totalCompatibility;

} GroupValue;

typedef struct st

{

int id;

string studentNumber;

int gender;

int hennyu;

} Student;

// Group information

typedef struct gn

{

int size; // Number of people in the group

int totalCompatibility; // Total compatibility

int minCompatibility; // Minimum compatibility

int numMinCompatibility; // Number of pairs with minimum compatibility

double evaluation; // Evaluation value

int numGirls; // Number of girls

int numBoys; // Number of boys

int numH; // Number of transfer students

bool feasible; // True if constraints are met

} GI;

// // Return a vector containing a random permutation on {0,...,n-1}

// vector<int> random_permutation(int n);

// /*** Read data from file ***/

// int read_file(const string &filename);

// /******************************************************************/

// /*** Add student i to group k ***/

// int joinStudent(int k, int i);

// /*** Remove student i from group k

// Returns 1 if the student exists, -1 if not ***/

// int removeStudent(int k, int i);

/*** Swap student s1 from group k1 with student s2 from group k2 ***/

/*** Returns 1 if the swap is successful, 0 if not ***/

int swap_students(int k1, int s1, int k2, int s2);

/*** Move student s1 from group k1 to group k2 ***/

/*** Returns 1 if successful, 0 if not ***/

int move_student(int k1, int s1, int k2);

/******************************************************************/

/*** make_initial_group: Create groups randomly ***/

void make_initial_group();

/*** Display list of information for group k ***/

void printGroup(int k);

/*** Print list of all groups ***/

void printAllGroup();

/*** Calculate evaluation value from compatibility ***/

double calc_evaluation(int s1, int s2);

/*** Evaluation value of group k ***/

//GroupValue evaluateGroup(int k);

/***

* gr[]: Array of students, gsize: Number of elements in the array

* Returns the evaluation value of the group

***/

double calc_group_evaluation(int *gr, int gsize);

// Update group information in groupInfo[MAX_NGROUP]

void update_groupInfo(int k);

/*********************************************************************/

// Return the difference in evaluation values after swapping student s1 from group k1 with student s2 from group k2

// Positive return value indicates an increase in evaluation value after the swap

double delta_by_swap(int k1, int s1, int k2, int s2);

// Return the difference in evaluation values after swapping (k1, s1) -> (k2, s2) -> (k3, s3)

// Positive return value indicates an increase in evaluation value after the swap

double delta_by_3swap(int k1, int s1, int k2, int s2, int k3, int s3);

/*** Simple local search ***/

int local_search_swap();

/*** Use 3swap to search for a better solution in the neighborhood

*** Try try3swap 3-neighborhood solutions

*** Move to the first found improved solution if first_find == true

*** Move to the best improved solution if first_find == false

*** Return 1 if there is such a neighborhood

*** Return 0 if there is none ***/

int local_search_by_3swap(int try3swap, bool first_find);

/*** Iterated local search ***/

double iterated_local_search(int **best_grouping, int *best_size, CRand &mt, int default_distance, int try3swap, bool first_find);

// Perform random 2-swap times times

void random_swap(int times);

#endif

## File “Makefile”

#CFLAGS=-g -Wall -std=c++11

CFLAGS= -Wall -std=c++11

OPT= -O

all: grouping_ils

grouping_ils: grouping_ils.o

c++ $(OPT) $(CFLAGS) -o grouping_ils grouping_ils.o

.cpp.o:

c++ $(CFLAGS) -c $<

clean:

/bin/rm -f grouping_ils *.o *~

## File “make_100_team_assignments.sh”

# This script let the team assignment program make 100 optimized results.

# grouping_ils : team assignment optimization program.

# data.txt : student data of properties and peer compatibility.

# results.txt : optimized team assignments.

# README for details.

echo `date '+%y/%m/%d %H:%M:%S'` > results.txt

for i in {0..99}

do

./grouping_ils data.txt >> results.txt

echo `date '+%y/%m/%d %H:%M:%S'` >> results.txt

echo "-----" >> results.txt

done

## File “data.txt”

64

0 1 0

1 0 0

2 0 0

3 1 0

4 1 0

5 0 0

6 0 0

7 0 0

8 0 0

9 1 0

10 0 0

11 1 0

12 0 0

13 0 0

14 0 0

15 1 0

16 1 0

17 0 0

18 1 0

19 1 0

20 0 0

21 0 0

22 1 0

23 0 0

24 1 0

25 1 0

26 0 0

27 0 0

28 0 0

29 1 0

30 1 0

31 0 0

32 1 0

33 0 0

34 0 0

35 0 0

36 1 0

37 1 0

38 1 0

39 0 0

40 1 0

41 0 0

42 0 0

43 1 0

44 0 0

45 0 0

46 0 0

47 0 0

48 0 0

49 0 0

50 0 0

51 1 0

52 0 0

53 0 0

54 0 0

55 1 0

56 0 1

57 1 1

58 0 1

59 0 1

60 0 1

61 0 1

62 1 1

63 0 1

0 1 5

0 2 4

0 3 7

0 4 5

0 5 7

0 6 7

0 7 5

0 8 5

0 9 1

0 10 4

0 11 5

0 12 5

0 13 10

0 14 7

0 15 10

0 16 7

0 17 4

0 18 10

0 19 8

0 20 6

0 21 5

0 22 7

0 23 4

0 24 1

0 25 7

0 26 7

0 27 2

0 28 3

0 29 4

0 30 9

0 31 8

0 32 9

0 33 5

0 34 6

0 35 4

0 36 3

0 37 9

0 38 3

0 39 8

0 40 10

0 41 7

0 42 1

0 43 4

0 44 1

0 45 8

0 46 8

0 47 1

0 48 2

0 49 6

0 50 7

0 51 6

0 52 3

0 53 1

0 54 4

0 55 1

0 56 10

0 57 5

0 58 1

0 59 2

0 60 2

0 61 2

0 62 4

0 63 6

1 2 5

1 3 10

1 4 4

1 5 8

1 6 7

1 7 3

1 8 4

1 9 10

1 10 5

1 11 1

1 12 1

1 13 2

1 14 6

1 15 10

1 16 2

1 17 3

1 18 4

1 19 1

1 20 2

1 21 3

1 22 4

1 23 2

1 24 3

1 25 8

1 26 3

1 27 3

1 28 5

1 29 5

1 30 1

1 31 5

1 32 7

1 33 1

1 34 5

1 35 3

1 36 1

1 37 4

1 38 10

1 39 5

1 40 7

1 41 4

1 42 10

1 43 3

1 44 3

1 45 8

1 46 4

1 47 4

1 48 6

1 49 10

1 50 1

1 51 7

1 52 9

1 53 10

1 54 3

1 55 4

1 56 3

1 57 3

1 58 5

1 59 3

1 60 5

1 61 5

1 62 8

1 63 5

2 3 4

2 4 1

2 5 5

2 6 2

2 7 7

2 8 9

2 9 10

2 10 1

2 11 1

2 12 5

2 13 1

2 14 2

2 15 10

2 16 10

2 17 1

2 18 6

2 19 4

2 20 7

2 21 1

2 22 7

2 23 5

2 24 2

2 25 9

2 26 1

2 27 3

2 28 9

2 29 8

2 30 3

2 31 6

2 32 1

2 33 6

2 34 8

2 35 1

2 36 6

2 37 7

2 38 1

2 39 3

2 40 4

2 41 1

2 42 4

2 43 1

2 44 8

2 45 2

2 46 4

2 47 3

2 48 8

2 49 4

2 50 1

2 51 8

2 52 3

2 53 5

2 54 8

2 55 5

2 56 4

2 57 5

2 58 8

2 59 3

2 60 1

2 61 1

2 62 9

2 63 9

3 4 2

3 5 1

3 6 1

3 7 3

3 8 5

3 9 5

3 10 4

3 11 5

3 12 1

3 13 7

3 14 2

3 15 5

3 16 1

3 17 4

3 18 7

3 19 4

3 20 2

3 21 7

3 22 4

3 23 5

3 24 10

3 25 4

3 26 1

3 27 3

3 28 2

3 29 7

3 30 5

3 31 10

3 32 6

3 33 3

3 34 4

3 35 6

3 36 9

3 37 1

3 38 5

3 39 3

3 40 4

3 41 1

3 42 6

3 43 8

3 44 10

3 45 1

3 46 4

3 47 5

3 48 1

3 49 1

3 50 6

3 51 4

3 52 4

3 53 7

3 54 7

3 55 7

3 56 1

3 57 4

3 58 5

3 59 9

3 60 1

3 61 6

3 62 2

3 63 3

4 5 3

4 6 10

4 7 5

4 8 5

4 9 1

4 10 7

4 11 4

4 12 5

4 13 6

4 14 10

4 15 3

4 16 6

4 17 8

4 18 8

4 19 1

4 20 4

4 21 1

4 22 5

4 23 1

4 24 2

4 25 5

4 26 2

4 27 3

4 28 9

4 29 8

4 30 5

4 31 8

4 32 1

4 33 1

4 34 5

4 35 4

4 36 1

4 37 9

4 38 1

4 39 4

4 40 6

4 41 4

4 42 7

4 43 8

4 44 10

4 45 1

4 46 1

4 47 4

4 48 3

4 49 1

4 50 10

4 51 7

4 52 7

4 53 4

4 54 10

4 55 7

4 56 1

4 57 7

4 58 4

4 59 4

4 60 3

4 61 9

4 62 5

4 63 8

5 6 8

5 7 6

5 8 10

5 9 4

5 10 7

5 11 10

5 12 7

5 13 4

5 14 6

5 15 8

5 16 10

5 17 7

5 18 2

5 19 8

5 20 6

5 21 2

5 22 4

5 23 7

5 24 8

5 25 2

5 26 4

5 27 4

5 28 10

5 29 4

5 30 1

5 31 5

5 32 5

5 33 8

5 34 6

5 35 6

5 36 3

5 37 3

5 38 2

5 39 7

5 40 1

5 41 5

5 42 8

5 43 4

5 44 8

5 45 8

5 46 10

5 47 3

5 48 9

5 49 3

5 50 1

5 51 4

5 52 7

5 53 1

5 54 3

5 55 10

5 56 6

5 57 4

5 58 3

5 59 7

5 60 2

5 61 6

5 62 8

5 63 1

6 7 7

6 8 1

6 9 3

6 10 10

6 11 4

6 12 10

6 13 3

6 14 5

6 15 3

6 16 3

6 17 5

6 18 5

6 19 1

6 20 7

6 21 2

6 22 1

6 23 6

6 24 4

6 25 5

6 26 10

6 27 1

6 28 7

6 29 2

6 30 3

6 31 10

6 32 4

6 33 1

6 34 5

6 35 1

6 36 6

6 37 4

6 38 1

6 39 5

6 40 8

6 41 10

6 42 9

6 43 7

6 44 6

6 45 1

6 46 5

6 47 7

6 48 5

6 49 5

6 50 5

6 51 5

6 52 3

6 53 1

6 54 8

6 55 4

6 56 4

6 57 4

6 58 9

6 59 7

6 60 3

6 61 1

6 62 8

6 63 9

7 8 6

7 9 5

7 10 1

7 11 7

7 12 5

7 13 5

7 14 5

7 15 5

7 16 7

7 17 10

7 18 1

7 19 5

7 20 6

7 21 10

7 22 1

7 23 1

7 24 8

7 25 1

7 26 9

7 27 1

7 28 3

7 29 3

7 30 4

7 31 9

7 32 2

7 33 5

7 34 6

7 35 3

7 36 7

7 37 9

7 38 10

7 39 10

7 40 5

7 41 6

7 42 8

7 43 6

7 44 6

7 45 9

7 46 5

7 47 10

7 48 7

7 49 1

7 50 2

7 51 3

7 52 4

7 53 2

7 54 5

7 55 6

7 56 3

7 57 4

7 58 7

7 59 4

7 60 3

7 61 2

7 62 4

7 63 6

8 9 1

8 10 5

8 11 5

8 12 2

8 13 1

8 14 5

8 15 8

8 16 7

8 17 4

8 18 3

8 19 7

8 20 4

8 21 5

8 22 2

8 23 4

8 24 7

8 25 9

8 26 6

8 27 10

8 28 7

8 29 3

8 30 4

8 31 7

8 32 8

8 33 1

8 34 1

8 35 6

8 36 4

8 37 10

8 38 6

8 39 3

8 40 4

8 41 9

8 42 3

8 43 1

8 44 3

8 45 6

8 46 5

8 47 3

8 48 1

8 49 4

8 50 1

8 51 6

8 52 6

8 53 7

8 54 5

8 55 3

8 56 3

8 57 5

8 58 5

8 59 9

8 60 8

8 61 3

8 62 7

8 63 3

9 10 7

9 11 7

9 12 5

9 13 5

9 14 6

9 15 9

9 16 5

9 17 3

9 18 3

9 19 3

9 20 5

9 21 5

9 22 2

9 23 5

9 24 5

9 25 10

9 26 4

9 27 6

9 28 5

9 29 5

9 30 5

9 31 1

9 32 6

9 33 4

9 34 1

9 35 7

9 36 3

9 37 10

9 38 3

9 39 4

9 40 6

9 41 1

9 42 8

9 43 4

9 44 6

9 45 5

9 46 1

9 47 5

9 48 4

9 49 7

9 50 2

9 51 5

9 52 8

9 53 10

9 54 7

9 55 1

9 56 6

9 57 3

9 58 2

9 59 3

9 60 10

9 61 1

9 62 5

9 63 2

10 11 5

10 12 10

10 13 3

10 14 3

10 15 10

10 16 4

10 17 5

10 18 5

10 19 10

10 20 3

10 21 4

10 22 1

10 23 2

10 24 2

10 25 1

10 26 6

10 27 1

10 28 8

10 29 5

10 30 6

10 31 8

10 32 10

10 33 8

10 34 4

10 35 7

10 36 8

10 37 4

10 38 4

10 39 5

10 40 2

10 41 6

10 42 8

10 43 5

10 44 8

10 45 5

10 46 2

10 47 6

10 48 10

10 49 3

10 50 4

10 51 3

10 52 1

10 53 5

10 54 7

10 55 5

10 56 5

10 57 7

10 58 1

10 59 1

10 60 4

10 61 8

10 62 1

10 63 3

11 12 2

11 13 3

11 14 5

11 15 10

11 16 3

11 17 4

11 18 10

11 19 1

11 20 7

11 21 2

11 22 10

11 23 8

11 24 4

11 25 5

11 26 3

11 27 5

11 28 1

11 29 5

11 30 8

11 31 1

11 32 3

11 33 3

11 34 1

11 35 7

11 36 8

11 37 6

11 38 1

11 39 10

11 40 5

11 41 1

11 42 7

11 43 1

11 44 10

11 45 5

11 46 1

11 47 5

11 48 2

11 49 5

11 50 7

11 51 2

11 52 8

11 53 3

11 54 1

11 55 2

11 56 3

11 57 6

11 58 9

11 59 6

11 60 3

11 61 5

11 62 7

11 63 2

12 13 5

12 14 5

12 15 1

12 16 8

12 17 2

12 18 10

12 19 1

12 20 6

12 21 6

12 22 6

12 23 3

12 24 9

12 25 6

12 26 7

12 27 4

12 28 6

12 29 7

12 30 7

12 31 8

12 32 1

12 33 6

12 34 7

12 35 9

12 36 2

12 37 6

12 38 8

12 39 9

12 40 9

12 41 2

12 42 2

12 43 4

12 44 1

12 45 2

12 46 2

12 47 7

12 48 8

12 49 1

12 50 2

12 51 6

12 52 5

12 53 1

12 54 6

12 55 2

12 56 1

12 57 4

12 58 9

12 59 10

12 60 4

12 61 1

12 62 7

12 63 2

13 14 1

13 15 6

13 16 4

13 17 10

13 18 1

13 19 3

13 20 6

13 21 3

13 22 3

13 23 4

13 24 1

13 25 1

13 26 6

13 27 4

13 28 1

13 29 3

13 30 10

13 31 8

13 32 2

13 33 3

13 34 1

13 35 8

13 36 1

13 37 7

13 38 6

13 39 3

13 40 7

13 41 6

13 42 10

13 43 8

13 44 8

13 45 4

13 46 2

13 47 2

13 48 9

13 49 2

13 50 3

13 51 3

13 52 3

13 53 1

13 54 2

13 55 7

13 56 9

13 57 7

13 58 5

13 59 8

13 60 4

13 61 5

13 62 5

13 63 4

14 15 1

14 16 2

14 17 1

14 18 3

14 19 9

14 20 3

14 21 7

14 22 1

14 23 4

14 24 3

14 25 7

14 26 5

14 27 7

14 28 4

14 29 3

14 30 1

14 31 4

14 32 1

14 33 4

14 34 4

14 35 10

14 36 7

14 37 1

14 38 4

14 39 1

14 40 1

14 41 5

14 42 6

14 43 4

14 44 6

14 45 4

14 46 3

14 47 3

14 48 6

14 49 10

14 50 1

14 51 10

14 52 1

14 53 6

14 54 1

14 55 6

14 56 7

14 57 4

14 58 10

14 59 2

14 60 6

14 61 4

14 62 7

14 63 8

15 16 5

15 17 4

15 18 3

15 19 4

15 20 3

15 21 6

15 22 9

15 23 1

15 24 4

15 25 5

15 26 4

15 27 5

15 28 10

15 29 5

15 30 4

15 31 5

15 32 4

15 33 2

15 34 1

15 35 6

15 36 6

15 37 4

15 38 6

15 39 7

15 40 4

15 41 4

15 42 4

15 43 1

15 44 9

15 45 5

15 46 9

15 47 6

15 48 4

15 49 3

15 50 5

15 51 9

15 52 6

15 53 8

15 54 3

15 55 5

15 56 5

15 57 2

15 58 5

15 59 10

15 60 5

15 61 4

15 62 4

15 63 2

16 17 1

16 18 6

16 19 1

16 20 7

16 21 8

16 22 2

16 23 2

16 24 1

16 25 6

16 26 8

16 27 5

16 28 3

16 29 5

16 30 6

16 31 2

16 32 1

16 33 3

16 34 10

16 35 4

16 36 1

16 37 8

16 38 5

16 39 2

16 40 1

16 41 4

16 42 9

16 43 6

16 44 5

16 45 9

16 46 6

16 47 4

16 48 4

16 49 7

16 50 2

16 51 6

16 52 5

16 53 6

16 54 9

16 55 3

16 56 7

16 57 8

16 58 4

16 59 3

16 60 5

16 61 10

16 62 2

16 63 8

17 18 7

17 19 3

17 20 1

17 21 7

17 22 5

17 23 6

17 24 9

17 25 7

17 26 1

17 27 4

17 28 5

17 29 7

17 30 5

17 31 5

17 32 4

17 33 7

17 34 1

17 35 1

17 36 7

17 37 8

17 38 6

17 39 1

17 40 1

17 41 1

17 42 5

17 43 3

17 44 3

17 45 3

17 46 3

17 47 3

17 48 7

17 49 10

17 50 6

17 51 2

17 52 6

17 53 6

17 54 7

17 55 8

17 56 8

17 57 3

17 58 3

17 59 3

17 60 3

17 61 1

17 62 8

17 63 8

18 19 8

18 20 7

18 21 6

18 22 4

18 23 5

18 24 3

18 25 1

18 26 1

18 27 6

18 28 7

18 29 6

18 30 1

18 31 6

18 32 2

18 33 5

18 34 4

18 35 5

18 36 6

18 37 2

18 38 6

18 39 6

18 40 1

18 41 4

18 42 8

18 43 3

18 44 10

18 45 5

18 46 3

18 47 5

18 48 8

18 49 6

18 50 9

18 51 2

18 52 7

18 53 6

18 54 1

18 55 9

18 56 9

18 57 7

18 58 8

18 59 5

18 60 4

18 61 2

18 62 5

18 63 2

19 20 7

19 21 4

19 22 3

19 23 4

19 24 8

19 25 3

19 26 6

19 27 5

19 28 7

19 29 4

19 30 6

19 31 7

19 32 1

19 33 4

19 34 6

19 35 6

19 36 3

19 37 10

19 38 7

19 39 5

19 40 2

19 41 5

19 42 4

19 43 7

19 44 3

19 45 5

19 46 2

19 47 3

19 48 5

19 49 2

19 50 4

19 51 5

19 52 4

19 53 1

19 54 1

19 55 5

19 56 5

19 57 7

19 58 8

19 59 5

19 60 8

19 61 5

19 62 9

19 63 10

20 21 9

20 22 5

20 23 3

20 24 10

20 25 5

20 26 1

20 27 6

20 28 4

20 29 5

20 30 4

20 31 2

20 32 4

20 33 8

20 34 6

20 35 4

20 36 8

20 37 4

20 38 1

20 39 7

20 40 2

20 41 5

20 42 1

20 43 1

20 44 7

20 45 2

20 46 7

20 47 1

20 48 2

20 49 1

20 50 3

20 51 3

20 52 4

20 53 8

20 54 1

20 55 5

20 56 1

20 57 8

20 58 5

20 59 5

20 60 3

20 61 3

20 62 8

20 63 10

21 22 7

21 23 6

21 24 4

21 25 2

21 26 5

21 27 6

21 28 9

21 29 9

21 30 3

21 31 7

21 32 3

21 33 5

21 34 1

21 35 6

21 36 1

21 37 5

21 38 6

21 39 6

21 40 1

21 41 8

21 42 5

21 43 1

21 44 1

21 45 5

21 46 5

21 47 6

21 48 10

21 49 4

21 50 7

21 51 4

21 52 5

21 53 6

21 54 10

21 55 6

21 56 5

21 57 10

21 58 2

21 59 7

21 60 1

21 61 6

21 62 5

21 63 7

22 23 6

22 24 3

22 25 4

22 26 1

22 27 7

22 28 1

22 29 2

22 30 6

22 31 6

22 32 4

22 33 1

22 34 3

22 35 3

22 36 4

22 37 7

22 38 4

22 39 5

22 40 3

22 41 2

22 42 2

22 43 4

22 44 1

22 45 4

22 46 3

22 47 1

22 48 7

22 49 4

22 50 4

22 51 7

22 52 5

22 53 6

22 54 5

22 55 8

22 56 8

22 57 6

22 58 7

22 59 8

22 60 5

22 61 1

22 62 6

22 63 4

23 24 5

23 25 2

23 26 3

23 27 4

23 28 7

23 29 3

23 30 1

23 31 1

23 32 2

23 33 10

23 34 6

23 35 2

23 36 2

23 37 2

23 38 4

23 39 2

23 40 4

23 41 6

23 42 9

23 43 3

23 44 1

23 45 6

23 46 4

23 47 2

23 48 5

23 49 8

23 50 4

23 51 4

23 52 2

23 53 9

23 54 4

23 55 1

23 56 1

23 57 4

23 58 3

23 59 3

23 60 4

23 61 5

23 62 4

23 63 3

24 25 4

24 26 5

24 27 6

24 28 3

24 29 1

24 30 1

24 31 2

24 32 9

24 33 6

24 34 6

24 35 7

24 36 6

24 37 6

24 38 2

24 39 3

24 40 2

24 41 10

24 42 3

24 43 7

24 44 3

24 45 1

24 46 3

24 47 7

24 48 1

24 49 1

24 50 1

24 51 5

24 52 2

24 53 4

24 54 4

24 55 3

24 56 6

24 57 9

24 58 6

24 59 3

24 60 5

24 61 1

24 62 2

24 63 5

25 26 5

25 27 5

25 28 4

25 29 10

25 30 1

25 31 6

25 32 1

25 33 3

25 34 7

25 35 2

25 36 4

25 37 10

25 38 4

25 39 3

25 40 5

25 41 2

25 42 8

25 43 7

25 44 2

25 45 7

25 46 5

25 47 5

25 48 1

25 49 3

25 50 1

25 51 4

25 52 10

25 53 5

25 54 5

25 55 2

25 56 1

25 57 3

25 58 10

25 59 9

25 60 6

25 61 6

25 62 8

25 63 1

26 27 3

26 28 3

26 29 4

26 30 5

26 31 5

26 32 2

26 33 1

26 34 1

26 35 5

26 36 4

26 37 3

26 38 5

26 39 2

26 40 6

26 41 2

26 42 1

26 43 4

26 44 5

26 45 1

26 46 6

26 47 7

26 48 5

26 49 3

26 50 8

26 51 7

26 52 2

26 53 1

26 54 5

26 55 4

26 56 6

26 57 5

26 58 5

26 59 2

26 60 6

26 61 5

26 62 5

26 63 4

27 28 1

27 29 2

27 30 1

27 31 3

27 32 1

27 33 1

27 34 6

27 35 1

27 36 3

27 37 1

27 38 5

27 39 5

27 40 3

27 41 8

27 42 6

27 43 1

27 44 7

27 45 3

27 46 4

27 47 7

27 48 5

27 49 7

27 50 3

27 51 7

27 52 3

27 53 7

27 54 3

27 55 3

27 56 5

27 57 7

27 58 1

27 59 5

27 60 4

27 61 5

27 62 5

27 63 4

28 29 5

28 30 8

28 31 6

28 32 4

28 33 3

28 34 2

28 35 5

28 36 5

28 37 6

28 38 9

28 39 4

28 40 6

28 41 6

28 42 1

28 43 8

28 44 2

28 45 5

28 46 5

28 47 6

28 48 3

28 49 1

28 50 4

28 51 5

28 52 5

28 53 6

28 54 7

28 55 4

28 56 1

28 57 4

28 58 1

28 59 8

28 60 5

28 61 10

28 62 1

28 63 7

29 30 2

29 31 2

29 32 2

29 33 6

29 34 2

29 35 6

29 36 1

29 37 1

29 38 4

29 39 3

29 40 8

29 41 5

29 42 9

29 43 6

29 44 7

29 45 4

29 46 5

29 47 3

29 48 1

29 49 1

29 50 1

29 51 5

29 52 1

29 53 10

29 54 5

29 55 7

29 56 3

29 57 4

29 58 5

29 59 4

29 60 4

29 61 5

29 62 1

29 63 2

30 31 3

30 32 1

30 33 3

30 34 5

30 35 8

30 36 7

30 37 3

30 38 5

30 39 4

30 40 8

30 41 6

30 42 10

30 43 1

30 44 1

30 45 1

30 46 6

30 47 6

30 48 3

30 49 1

30 50 2

30 51 3

30 52 5

30 53 7

30 54 6

30 55 8

30 56 1

30 57 3

30 58 7

30 59 7

30 60 2

30 61 6

30 62 4

30 63 6

31 32 9

31 33 1

31 34 6

31 35 4

31 36 8

31 37 8

31 38 10

31 39 5

31 40 3

31 41 1

31 42 1

31 43 4

31 44 9

31 45 1

31 46 4

31 47 5

31 48 6

31 49 8

31 50 5

31 51 3

31 52 3

31 53 1

31 54 7

31 55 7

31 56 1

31 57 1

31 58 1

31 59 4

31 60 1

31 61 10

31 62 2

31 63 8

32 33 6

32 34 5

32 35 1

32 36 5

32 37 3

32 38 5

32 39 6

32 40 8

32 41 1

32 42 1

32 43 7

32 44 2

32 45 1

32 46 1

32 47 1

32 48 1

32 49 4

32 50 1

32 51 7

32 52 4

32 53 4

32 54 2

32 55 2

32 56 10

32 57 3

32 58 6

32 59 4

32 60 7

32 61 7

32 62 3

32 63 8

33 34 7

33 35 2

33 36 6

33 37 2

33 38 1

33 39 7

33 40 5

33 41 3

33 42 1

33 43 1

33 44 3

33 45 4

33 46 1

33 47 2

33 48 1

33 49 2

33 50 6

33 51 8

33 52 1

33 53 2

33 54 1

33 55 10

33 56 8

33 57 9

33 58 6

33 59 1

33 60 9

33 61 7

33 62 7

33 63 7

34 35 4

34 36 3

34 37 4

34 38 1

34 39 1

34 40 1

34 41 5

34 42 2

34 43 4

34 44 6

34 45 7

34 46 7

34 47 3

34 48 4

34 49 5

34 50 5

34 51 8

34 52 2

34 53 5

34 54 4

34 55 8

34 56 2

34 57 7

34 58 7

34 59 2

34 60 7

34 61 4

34 62 4

34 63 2

35 36 5

35 37 5

35 38 4

35 39 1

35 40 5

35 41 4

35 42 2

35 43 5

35 44 1

35 45 5

35 46 2

35 47 1

35 48 2

35 49 4

35 50 2

35 51 2

35 52 6

35 53 7

35 54 8

35 55 7

35 56 6

35 57 4

35 58 7

35 59 3

35 60 4

35 61 3

35 62 1

35 63 4

36 37 8

36 38 10

36 39 1

36 40 9

36 41 5

36 42 1

36 43 9

36 44 4

36 45 5

36 46 1

36 47 2

36 48 9

36 49 9

36 50 4

36 51 1

36 52 5

36 53 4

36 54 9

36 55 4

36 56 5

36 57 6

36 58 1

36 59 7

36 60 3

36 61 2

36 62 8

36 63 5

37 38 1

37 39 4

37 40 2

37 41 1

37 42 6

37 43 3

37 44 6

37 45 1

37 46 10

37 47 3

37 48 2

37 49 1

37 50 1

37 51 5

37 52 10

37 53 10

37 54 1

37 55 8

37 56 2

37 57 8

37 58 10

37 59 1

37 60 6

37 61 3

37 62 5

37 63 10

38 39 5

38 40 3

38 41 3

38 42 10

38 43 10

38 44 1

38 45 5

38 46 5

38 47 5

38 48 3

38 49 8

38 50 1

38 51 1

38 52 7

38 53 3

38 54 1

38 55 4

38 56 2

38 57 2

38 58 7

38 59 1

38 60 2

38 61 7

38 62 10

38 63 7

39 40 1

39 41 7

39 42 1

39 43 2

39 44 2

39 45 10

39 46 3

39 47 5

39 48 1

39 49 1

39 50 5

39 51 8

39 52 5

39 53 4

39 54 2

39 55 1

39 56 9

39 57 8

39 58 7

39 59 4

39 60 1

39 61 4

39 62 6

39 63 7

40 41 3

40 42 8

40 43 10

40 44 9

40 45 1

40 46 2

40 47 4

40 48 5

40 49 4

40 50 2

40 51 1

40 52 2

40 53 1

40 54 5

40 55 2

40 56 1

40 57 5

40 58 6

40 59 2

40 60 3

40 61 7

40 62 1

40 63 7

41 42 3

41 43 1

41 44 5

41 45 6

41 46 5

41 47 3

41 48 7

41 49 9

41 50 4

41 51 1

41 52 9

41 53 6

41 54 6

41 55 7

41 56 10

41 57 1

41 58 3

41 59 1

41 60 8

41 61 10

41 62 8

41 63 4

42 43 10

42 44 2

42 45 2

42 46 4

42 47 7

42 48 1

42 49 6

42 50 7

42 51 5

42 52 5

42 53 1

42 54 7

42 55 6

42 56 9

42 57 1

42 58 7

42 59 9

42 60 1

42 61 6

42 62 3

42 63 9

43 44 7

43 45 5

43 46 4

43 47 5

43 48 7

43 49 1

43 50 5

43 51 2

43 52 5

43 53 3

43 54 1

43 55 6

43 56 7

43 57 2

43 58 1

43 59 3

43 60 5

43 61 10

43 62 3

43 63 5

44 45 4

44 46 9

44 47 7

44 48 5

44 49 3

44 50 6

44 51 6

44 52 7

44 53 5

44 54 6

44 55 3

44 56 4

44 57 2

44 58 1

44 59 7

44 60 7

44 61 3

44 62 5

44 63 10

45 46 5

45 47 4

45 48 1

45 49 8

45 50 1

45 51 1

45 52 4

45 53 10

45 54 1

45 55 10

45 56 3

45 57 1

45 58 2

45 59 6

45 60 4

45 61 4

45 62 9

45 63 4

46 47 7

46 48 3

46 49 3

46 50 5

46 51 1

46 52 1

46 53 7

46 54 6

46 55 9

46 56 5

46 57 6

46 58 6

46 59 10

46 60 8

46 61 1

46 62 5

46 63 8

47 48 3

47 49 9

47 50 8

47 51 1

47 52 3

47 53 1

47 54 6

47 55 4

47 56 4

47 57 5

47 58 7

47 59 5

47 60 5

47 61 3

47 62 2

47 63 2

48 49 5

48 50 6

48 51 3

48 52 8

48 53 2

48 54 10

48 55 3

48 56 7

48 57 4

48 58 2

48 59 8

48 60 10

48 61 5

48 62 8

48 63 1

49 50 6

49 51 6

49 52 6

49 53 6

49 54 2

49 55 5

49 56 3

49 57 1

49 58 9

49 59 2

49 60 3

49 61 2

49 62 7

49 63 6

50 51 7

50 52 5

50 53 4

50 54 1

50 55 5

50 56 2

50 57 8

50 58 2

50 59 10

50 60 1

50 61 6

50 62 2

50 63 6

51 52 6

51 53 1

51 54 2

51 55 7

51 56 5

51 57 4

51 58 6

51 59 4

51 60 8

51 61 1

51 62 2

51 63 4

52 53 7

52 54 1

52 55 1

52 56 10

52 57 2

52 58 6

52 59 3

52 60 3

52 61 4

52 62 2

52 63 6

53 54 4

53 55 3

53 56 7

53 57 3

53 58 5

53 59 4

53 60 1

53 61 2

53 62 6

53 63 2

54 55 2

54 56 5

54 57 6

54 58 5

54 59 5

54 60 5

54 61 1

54 62 10

54 63 1

55 56 2

55 57 7

55 58 5

55 59 5

55 60 3

55 61 4

55 62 1

55 63 1

56 57 3

56 58 5

56 59 9

56 60 1

56 61 4

56 62 2

56 63 6

57 58 5

57 59 1

57 60 5

57 61 7

57 62 6

57 63 5

58 59 5

58 60 4

58 61 3

58 62 8

58 63 2

59 60 7

59 61 3

59 62 6

59 63 4

60 61 10

60 62 2

60 63 6

61 62 9

61 63 8

62 63 4

0 0 0

## File results.txt

24/05/23 17:00:11

data.txt

0,4,3g,29g,44,53,7 10 7 7 10 5 ,44.0,46,2,2,0,

1,4,14,19g,58H,62gH,9 10 7 8 9 8 ,51.0,51,2,2,2,

2,4,46,47,50,59H,7 5 10 8 5 10 ,41.0,45,0,4,1,

3,4,5,23,33,34,7 8 6 10 6 7 ,44.0,44,0,4,0,

4,4,18g,36g,45,49,6 5 6 5 9 8 ,35.0,39,2,2,0,

5,4,39,41,52,56H,7 5 9 9 10 10 ,48.0,50,0,4,1,

6,4,6,10,12,26,10 10 10 10 6 7 ,53.0,53,0,4,0,

7,4,1,2,9g,25g,5 10 8 10 9 10 ,50.0,52,2,2,0,

8,4,17,21,48,54,7 7 7 10 10 10 ,51.0,51,0,4,0,

9,4,8,32g,51g,60H,8 6 8 7 7 8 ,44.0,44,2,2,1,

10,4,28,40g,43g,61H,6 8 10 10 7 10 ,51.0,51,2,2,1,

11,4,0g,11g,15g,22g,5 10 7 10 10 9 ,49.0,51,4,0,0,

12,4,7,16g,38g,42,7 10 8 5 9 10 ,47.0,49,2,2,0,

13,4,13,30g,35,55g,10 8 7 8 8 7 ,48.0,48,2,2,0,

14,4,20,24g,27,57gH,10 6 8 6 9 7 ,46.0,46,2,2,1,

15,4,4g,31,37g,63H,8 9 8 8 8 10 ,51.0,51,2,2,1,

Total Value = 753.0

Minimum Compatibility = 5

Total Compatibility = 771

24/05/23 17:05:21

-----

data.txt

0,4,7,26,47,58H,9 10 7 7 5 7 ,43.0,45,0,4,1,

1,4,8,19g,24g,60H,7 7 8 8 8 5 ,41.0,43,2,2,1,

2,4,11g,15g,50,59H,10 7 6 5 10 10 ,46.0,48,2,2,1,

3,4,5,20,34,46,6 6 10 6 7 7 ,42.0,42,0,4,0,

4,4,36g,48,54,62gH,9 9 8 10 8 10 ,54.0,54,2,2,1,

5,4,0g,32g,39,56H,9 8 10 6 10 9 ,52.0,52,2,2,1,

6,4,3g,29g,35,53,7 6 7 6 10 7 ,43.0,43,2,2,0,

7,4,21,22g,27,57gH,7 6 10 7 6 7 ,43.0,43,2,2,1,

8,4,17,18g,33,55g,7 7 8 5 9 10 ,44.0,46,2,2,0,

9,4,6,10,12,31,10 10 10 10 8 8 ,56.0,56,0,4,0,

10,4,13,30g,40g,42,10 7 10 8 10 8 ,53.0,53,2,2,0,

11,4,2,16g,37g,63H,10 7 9 8 8 10 ,52.0,52,2,2,1,

12,4,1,9g,25g,52,10 8 9 10 8 10 ,55.0,55,2,2,0,

13,4,4g,14,44,51g,10 10 7 6 10 6 ,49.0,49,2,2,0,

14,4,28,38g,43g,61H,9 8 10 10 7 10 ,54.0,54,2,2,1,

15,4,23,41,45,49,6 6 8 6 9 8 ,43.0,43,0,4,0,

Total Value = 770.0

Minimum Compatibility = 5

Total Compatibility = 778

24/05/23 17:10:25

-----

……There are 98 more outputs to follow.
