# Supplementary material for: Optimizing anatomy dissection teams using the Yukari method: A peer compatibility‐based approach
Source: Anat Sci Educ. 2025 Oct 3;18(11):1262–77. doi: 10.1002/ase.70124 (PMC12592916; doi:10.1002/ase.70124)
Supplement: Supplementary file 3 — Appendix A3. Yukari viewpoint survey. This appendix provides the full text of the Yukari Viewpoint Survey, which was administered to anatomy students after the completion of the course. It was designed to evaluate students’ impressions and satisfaction with their team and the assignment method. [file ASE-18-1262-s009.docx]

# APPENDIX A3: Yukari Viewpoint Survey

The following survey is implemented on a secure Google Form (Google, Mountain View, CA) and is administered to all students after the completion of the anatomy course for the evaluation of team assignments.

## Anatomy Team Viewpoint Survey

We, the anatomy faculty, are committed to improving the method of assigning dissection teams. Please share your viewpoints regarding your dissection team experience.

Only one faculty member, bound by confidentiality, will handle the data. The data will be used exclusively to improve the team assignment process. Anonymized statistical data from this survey may be disclosed in scientific reports.

* Required questions

### I thought my own team was good when the assignment was announced before the dissection started. *

Choose the single option that best reflects your agreement with the statement above.

- Agree
- Somewhat agree
- Neither agree nor disagree
- Somewhat disagree
- Disagree

### I thought my team was eventually good when the anatomy class was completed. *

Choose the single option that best reflects your agreement with the statement above.

- Agree
- Somewhat agree
- Neither agree nor disagree
- Somewhat disagree
- Disagree

### My team assignment method was good. *

Choose the single option that best reflects your agreement with the statement above.

- Agree
- Somewhat agree
- Neither agree nor disagree
- Somewhat disagree
- Disagree

### Please write any comments regarding your dissection team and its assignment.

[ ]

#### [SUBMIT BOTTON]
