# Supplementary figures and images for: Optimizing anatomy dissection teams using the Yukari method: A peer compatibility‐based approach
Source: Anat Sci Educ. 2025 Oct 3;18(11):1262–77. doi: 10.1002/ase.70124 (PMC12592916; doi:10.1002/ase.70124)

Figure S1

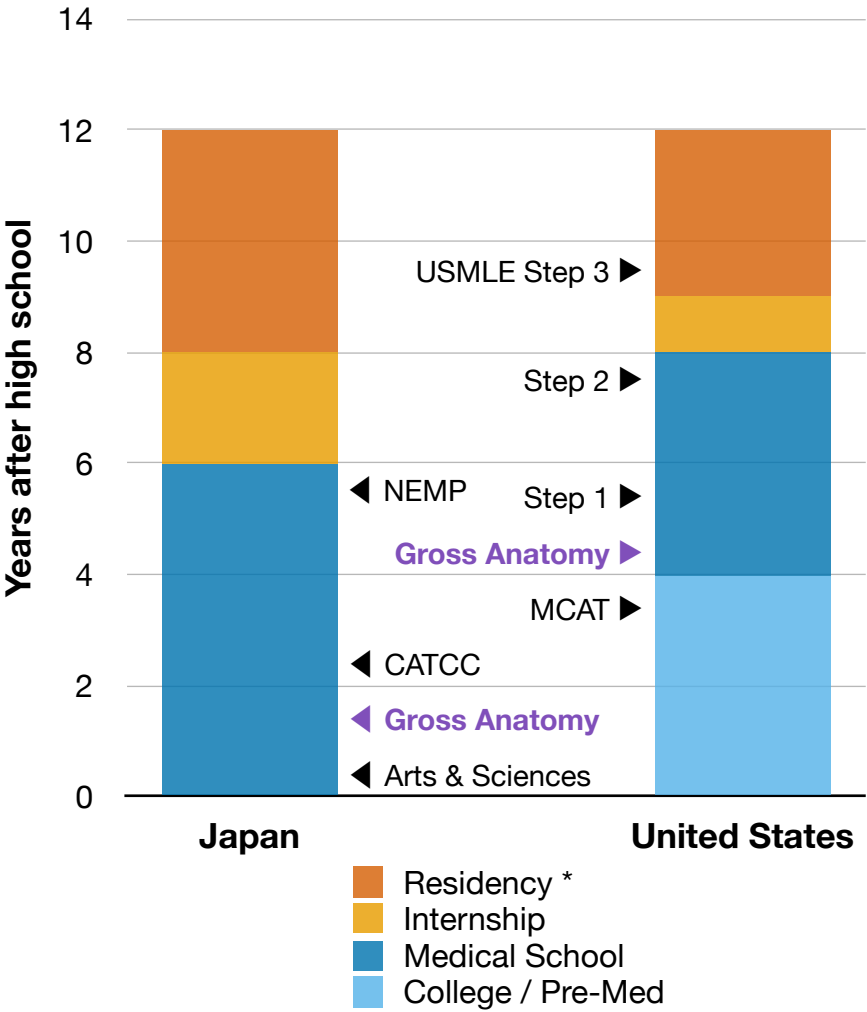

Supplement: Supplementary file 4 — Figure S1. Comparison of medical education in Japan and the United States up to residency. The medical education systems in Japan and the United States have been summarized and aligned in a diagram for comparison. Equivalent processes are indicated by bars of the same color. The vertical axis represents the number of years post‐high school graduation. NEMP = National Examination for Medical Practitioners (Japan). CATCC = Common Achievement Tests to Clinical Clerkship, followed by clinical clerkship at hospitals (Japan). USMLE = US Medical Licensing Examination. MCAT = Medical College Admission Test (USA). * Residency duration varies depending on the program. [file ASE-18-1262-s001.pdf]

Figure S2

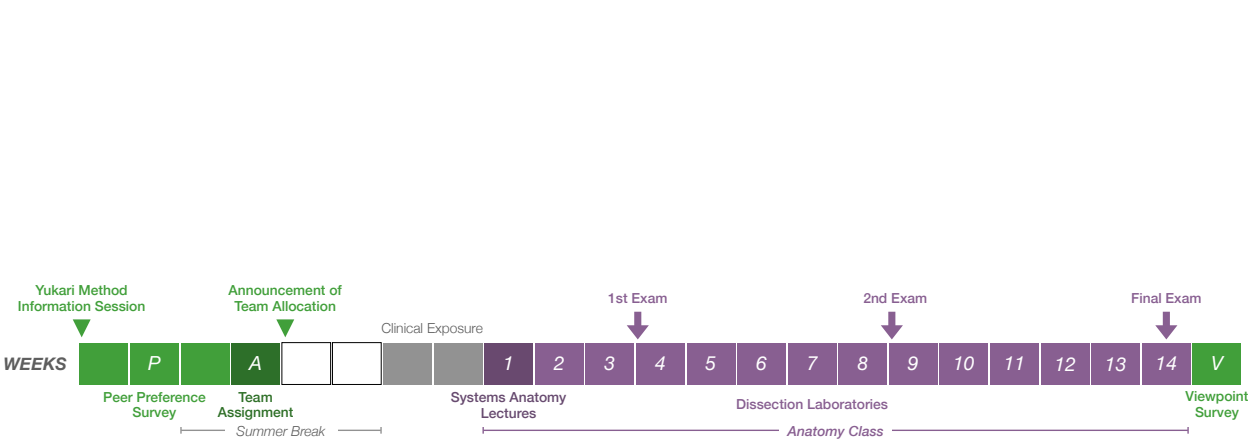

Supplement: Supplementary file 5 — Figure S2. Weekly timeline integrating the Yukari method with the gross anatomy course. A schematic weekly timeline illustrating incorporation of the Yukari method (green) into the gross anatomy course (purple). The diagram shows the sequence of key events, including the information session, peer preference survey, team assignment, anatomy lectures, dissection laboratories, surveys, and exams, highlighting integration of team formation with the anatomy course. [file ASE-18-1262-s004.pdf]

Figure S3

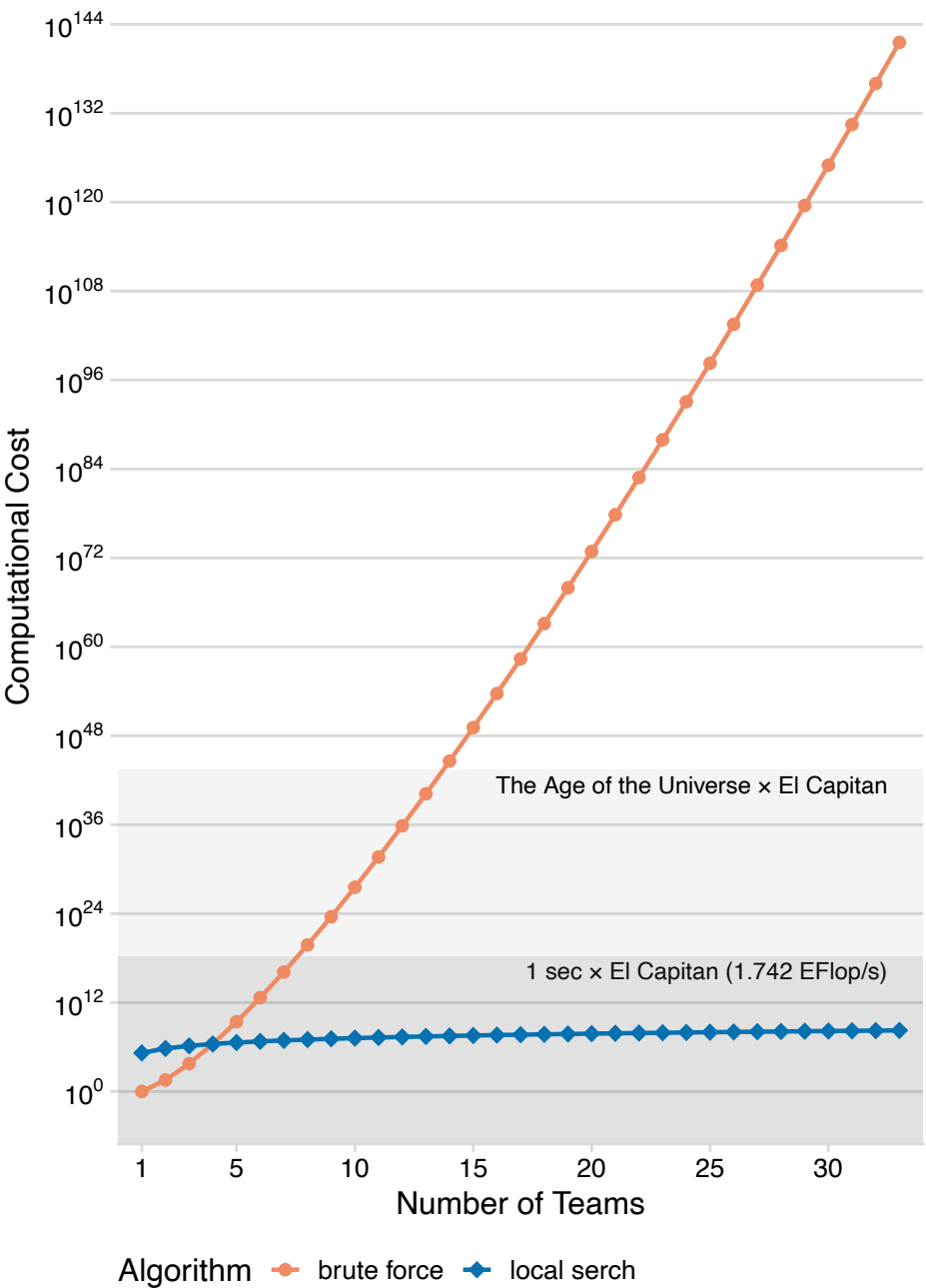

Supplement: Supplementary file 6 — Figure S3. Computational cost of brute‐force and local search team assignment optimization. Computational costs of team assignment optimization were compared between brute‐force (red) and local search (blue). The evaluation assumes teams of 4 members each. The shaded regions indicate the computational cost by El Capitan, one of the fastest supercomputers as of June 2025, with a peak speed at 1.742 EFlop/s. The x‐axis shows class size (number of teams), and the y‐axis shows computational cost (log scale, base 10). [file ASE-18-1262-s006.pdf]

Figure S4

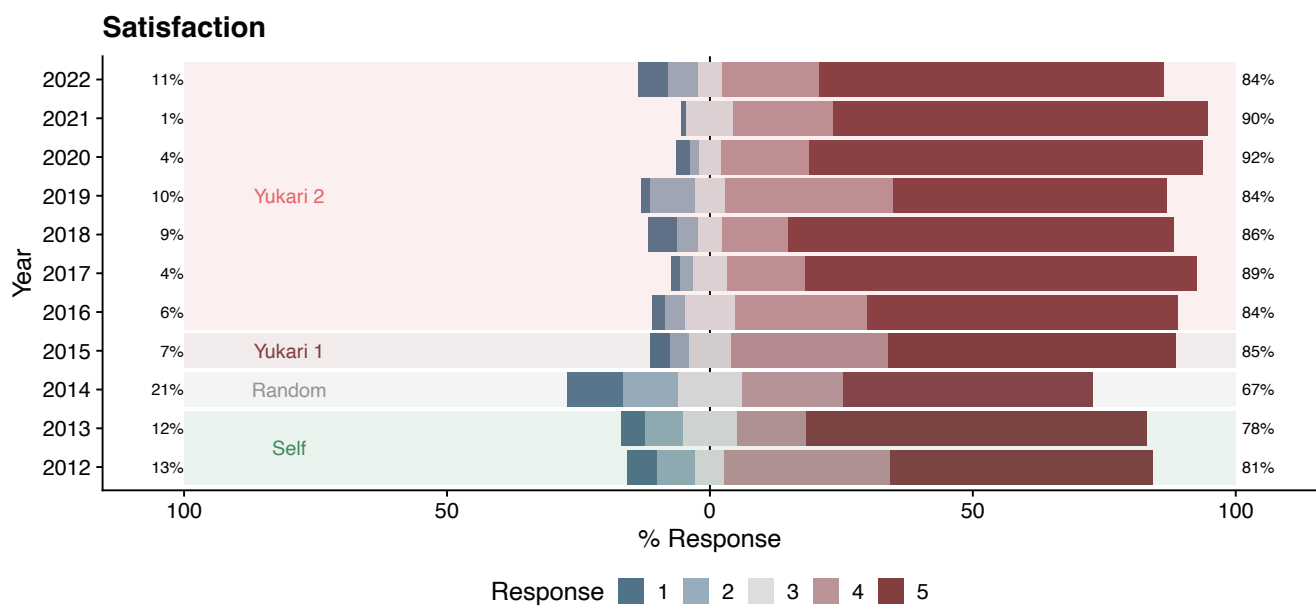

Supplement: Supplementary file 7 — Figure S4. Trends of students’ satisfaction with teams over the COVID‐19 pandemic. Students’ satisfaction on a Likert scale (1–5; higher is better) from 2012 to 2022 is summarized as a Likert plot. Note that high levels of student satisfaction were maintained over the COVID‐19 pandemic. [file ASE-18-1262-s008.pdf]
